# CircIL4R activates the PI3K/AKT signaling pathway via the miR-761/TRIM29/PHLPP1 axis and promotes proliferation and metastasis in colorectal cancer

**DOI:** 10.1186/s12943-021-01474-9

**Published:** 2021-12-18

**Authors:** Tao Jiang, Hongyu Wang, Lianyu Liu, Hu Song, Yi Zhang, Jiaqi Wang, Lei Liu, Teng Xu, Ruizhi Fan, Yixin Xu, Shuai Wang, Linsen Shi, Li Zheng, Renhao Wang, Jun Song

**Affiliations:** 1 Department of General Surgery, The Affiliated Hospital of Xuzhou Medical University, Xuzhou, 221006 Jiangsu China; 2 Institute of Digestive Diseases, Xuzhou Medical University, Xuzhou, 221002 Jiangsu China; 3 The Graduate School, Xuzhou Medical University, Xuzhou, 221004 Jiangsu China; 4 State Key Laboratory of Proteomics, Beijing Proteome Research Center, National Center for Protein Sciences, Beijing Institute of Lifeomics, Beijing, China

**Keywords:** circIL4R, miR-761, TRIM29, PI3K/AKT pathway, Colorectal cancer

## Abstract

**Background:**

Accumulating studies have revealed that aberrant expression of circular RNAs (circRNAs) is widely involved in the tumorigenesis and progression of malignant cancers, including colorectal cancer (CRC). Nevertheless, the clinical significance, levels, features, biological function, and molecular mechanisms of novel circRNAs in CRC remain largely unexplored.

**Methods:**

CRC-related circRNAs were identified through bioinformatics analysis and verified in clinical specimens by qRT–PCR and in situ hybridization (ISH). Then, in vitro and in vivo experiments were performed to determine the clinical significance of, functional roles of, and clinical characteristics associated with circIL4R in CRC specimens and cells. Mechanistically, RNA pull-down, fluorescence in situ hybridization (FISH), luciferase reporter, and ubiquitination assays were performed to confirm the underlying mechanism of circIL4R.

**Results:**

CircIL4R was upregulated in CRC cell lines and in sera and tissues from CRC patients and was positively correlated with advanced clinicopathological features and poor prognosis. Functional experiments demonstrated that circIL4R promotes CRC cell proliferation, migration, and invasion via the PI3K/AKT signaling pathway. Mechanistically, circIL4R was regulated by TFAP2C and competitively interacted with miR-761 to enhance the expression of TRIM29, thereby targeting PHLPP1 for ubiquitin-mediated degradation to activate the PI3K/AKT signaling pathway and consequently facilitate CRC progression.

**Conclusions:**

Our findings demonstrate that upregulation of circIL4R plays an oncogenic role in CRC progression and may serve as a promising diagnostic and prognostic biomarker for CRC detection and as a potential therapeutic target for CRC treatment.

**Supplementary Information:**

The online version contains supplementary material available at 10.1186/s12943-021-01474-9.

## Background

Colorectal cancer (CRC), one of the most common malignancies of the digestive system, is the second leading cause of cancer-related death in the world [1]. National Comprehensive Cancer Network (NCCN) guidelines recommend surgery combined with radiotherapy and chemotherapy as the standard treatment for CRC [2]. Although CRC treatment has improved, the prognosis of CRC unfortunately remains unsatisfactory, primarily due to the unclear tumorigenesis mechanism and aggressive nature of CRC; approximately 880,000 CRC patients die annually [3]. Thus, it is urgent to understand and explore the underlying mechanism of the malignant progression of CRC to improve diagnostic efficiency, treatment effect and prognosis.

Circular RNAs (circRNAs), a novel class of bifunctional RNAs that possess noncoding and limited protein-coding functions, are generated from the noncanonical back-splicing junction site of precursor mRNAs and characterized by a covalently closed single-stranded loop structure [4]. Due to their lack of a 5′ cap and 3′ poly-A tail, circRNAs are resistant to exonucleases and more stable than their linear RNA counterparts [5]. Moreover, with the development of high-throughput sequencing combined with available bioinformatics analysis technology, accumulating evidence has revealed that circRNAs are widely expressed, generally stable, highly conserved and tissue- or cell-specific in malignant cancers, including CRC [6, 7]. For instance, the circRNA ciRS-7-A, circNSUN2, and circ-ERBIN were reported to be overexpressed in CRC and facilitate its progression [8–10]. circRNAs can exert their biological functions by acting as microRNA (miRNA) sponges and interacting with RNA-binding proteins (RBPs). Additionally, some circRNAs can function as translation templates or scaffolds of proteins or regulators of RNA splicing and gene transcription [11–13]. Aberrantly expressed circRNAs also play important roles in mediating cancer progression by regulating the activity of a variety of signaling pathways, such as the WNT/β-catenin, MAPK, JAK/STAT, NOTCH and PI3K/AKT pathways [14, 15]. For example, Zhang et al. revealed that hsa_circ_0026628 facilitates the progression of CRC by targeting SP1 to activate the Wnt/β-catenin signaling pathway [16]. Zheng et al. reported that circPPP1R12A promotes the pathogenesis and metastasis of colon cancer via the Hippo-YAP signaling pathway [17]. In summary, circRNAs may serve as potential biomarkers for CRC diagnosis and prognosis prediction, as well as effective therapeutic targets and vital regulators of signaling pathways [18].

TRIM29 (also known as ATDC), a member of the tripartite motif protein family, has been shown to play oncogenic roles in various cancers, including pancreatic cancer, thyroid cancer, bladder cancer, and CRC [19–24]. Sun et al. and Han et al. demonstrated that TRIM29 is upregulated in CRC and facilitates cancer progression [22, 23]. Xu et al. also reported that TRIM29 overexpression can promote thyroid cancer progression by activating the PI3K/AKT signaling pathway [24]. Recently, accumulating studies have confirmed that circRNAs play a role in the tumorigenesis and progression of multiple malignancies by modulating their target genes [9, 10]. Therefore, investigating the relationship between circRNAs and TRIM29 is of great significance for exploring the upstream regulation of TRIM29 and developing promising therapeutic targets for CRC.

In recent years, the aberrant expression and regulatory axes of circRNAs have been elucidated and described in a variety of malignancies. Nevertheless, only preliminary studies have been conducted on the role of circRNAs in CRC, and the clinical significance, levels, biological function, and molecular mechanisms of novel circRNAs in CRC remain largely unexplored. In the present study, based on bioinformatic analysis, we identified a novel CRC-related circRNA (hsa_circ_0038718) derived from exons 3 and 4 of the IL4R gene, also known as circIL4R. The expression and function of circIL4R in CRC was unclear. Hence, in situ hybridization (ISH) and qRT–PCR assays were first performed to determine the levels of circIL4R and the clinical significance of its expression. Subsequently, gain- and loss-of-function experiments were conducted in vitro and in vivo to identify the biological function and molecular mechanisms of circIL4R in CRC. More precisely, a series of experiments, such as chromatin immunoprecipitation (ChIP), luciferase reporter and biotinylated RNA pull-down assays, revealed that TFAP2C-induced circIL4R can act as a sponge for miR-761 to enhance TRIM29 expression, and forced overexpression of TRIM29 promotes ubiquitin-mediated degradation of PHLPP1 to activate the PI3K/AKT signaling pathway and promote the proliferation and metastasis of CRC cells. Collectively, our findings demonstrated that circIL4R may serve as a novel diagnostic and prognostic biomarker as well as a promising therapeutic target for CRC.

## Materials and methods

### Clinical specimens

A cohort comprising 120 pairs of CRC tissues and corresponding adjacent normal tissues (ANTs) was collected from CRC patients who underwent radical resection or palliative resection between January 2015 and September 2016, and the detailed clinicopathological features are summarized and analyzed in Additional file 1: Table S1. Another cohort consisted of 50 patients with diagnosed CRC; preoperative and postoperative serum samples collected from these patients before and after surgery between January 2019 and January 2020. We also obtained serum from 40 healthy subjects and 40 newly diagnosed CRC patients at the same time. In addition, a cohort comprising 179 pairs of paraffin-embedded CRC tissues and corresponding ANTs was collected to conduct the tissue microarrays (TMAs). All the CRC patients had pathologically confirmed disease and received no radiotherapy or chemotherapy before surgery. The specimens were collected by researchers of the General Surgery Department of the Affiliated Hospital of Xuzhou Medical University, immediately transported in liquid nitrogen after excision and eventually stored at − 80 °C prior to RNA extraction. This study was reviewed and approved by the Ethics Committee of the Affiliated Hospital of Xuzhou Medical University (XYFY2020-KL185–01), and the patients and control subjects provided informed consent.

### Cell lines and culture

The human normal colorectal epithelial cell line FHC was obtained from American Type Culture Collection (Manassas, VA, USA), while several human CRC cell lines (HCT116, DLD1, LoVo, SW620, HT29 and SW480) were obtained from the Cell Bank of the Chinese Academy of Sciences (Shanghai, China). HCT116 cells were cultured in McCoy’s 5A medium supplemented with 10% fetal bovine serum (FBS) (Gibco, USA), while SW620 cells were cultured in L-15 medium (Gibco, USA). DLD1, HT-29, and LoVo cells were cultured in RPMI 1640 medium (Gibco, USA), and FHC and SW480 cells were cultured in DMEM/high-glucose medium (Gibco, USA). All the cells were cultured in a constant temperature incubator at 37 °C containing 5%CO_2_ and subjected to mycoplasma testing to confirm that they were free of infection before use.

### Cell transfection

siRNAs targeting circIL4R (sicircIL4R#1, sicircIL4R#2), TRIM29 (siTRIM29), TFAP2C (siTFAP2C), and PHLPP1 (siPHLPP1); negative control (NC), siRNA (siCtrl); miR-761 mimics and inhibitor, miR-541-3p mimics and their respective NCs were designed and synthesized by Gene Pharma Technology (Shanghai, China). The cells were cultured to 30–50% confluence before they were transfected with the siRNAs by siLentFect Lipid Reagent (Bio-Rad, CA, USA) according to the to the manufacturer’s instructions. To construct overexpression plasmids, the full-length sequences of circIL4R, TRIM29, PHLPP1 and TFAP2C were cloned into the pcDNA3.1 vector (Invitrogen, Shanghai, China), while empty vector was used as a negative control. When cells reached approximately 90% confluence, they were transfected with the plasmids by Lipofectamine 2000 (Invitrogen, Shanghai, China) according to the manufacturer’s instructions. The transfected cells were harvested for subsequent experiments 48 h later. Lentiviruses against human circIL4R (sh-circIL4R#1 and sh-circIL4R#2) and nonspecific control lentiviruses (sh-Ctrl) were synthesized by Gene Pharma Technology (Shanghai, China) and transfected into CRC cells according to the manufacturer’s instructions. The CRC cells with stable circIL4R knockdown were selected with puromycin and verified by qRT–PCR. The sequences of the siRNA, shRNA and plasmids are described in the Additional file 1: Table S2.

### RNA, genomic DNA (gDNA) extraction and qRT–PCR

Total RNA was isolated from CRC specimens and cells using RNA Isolater Total RNA Extraction Reagent (Vazyme, Nanjing, China), whereas gDNA was extracted using a FastPure® Cell/Tissue DNA Isolation Mini Kit (Vazyme, Nanjing, China) according to the manufacturer’s guidelines. Nuclear and cytoplasmic RNA fractions from CRC cell lines were extracted using a PARIS™ Kit (Life Technologies, Austin, Texas, USA). The quality and concentration of the extracted samples were determined by a Nanodrop 2000 (Thermo Fisher Scientific, USA). For qRT–PCR analysis of circRNA and mRNA expression, RNA was first reverse-transcribed to cDNA using HiScript II Q RT SuperMix (Vazyme, Nanjing, China), which was then detected by ChamQ SYBR qPCR Master Mix (Vazyme, Nanjing, China) on a LightCycler 96 Instrument (Roche, Switzerland) using the following thermal conditions: 95 °C for 30 s; 40 cycles at 95 °C for 10 s and 60 °C for 60 s; and a melting curve analysis. For qRT–PCR analysis of miRNA, reverse transcription was performed using the miRNA 1st Strand cDNA Synthesis Kit (Vazyme, Nanjing, China) with specific stem-loop primers, and qRT–PCR was then performed with miRNA Universal SYBR qPCR Master Mix (Vazyme, Nanjing, China) on a LightCycler 96 Instrument using the following cycling conditions: 95 °C for 5 min; 40 cycles at 95 °C for 10 s and 60 °C for 60 s; and a melting curve analysis. The relative RNA expression level was calculated using the 2^-ΔΔCT^ method, 18S rRNA serving as a reference for circRNAs, GAPDH serving as a reference for mRNAs, and U6 serving as a reference for miRNAs. The sequences of the primers used are listed in Additional file 1: Table S3.

### Chromatin immunoprecipitation (ChIP) assay

The ChIP assay was performed using an EZ ChIP Chromatin Immunoprecipitation Kit (Millipore, Bedford, MA, USA) according to the manufacturer’s guidelines. Briefly, HCT116 cells (1 × 10^7^) were treated with 1% formaldehyde at room temperature for 10 min to crosslink the DNA and proteins, after which they were washed with PBS and lysed with ChIP lysis buffer. Subsequently, the lysates were sonicated to obtain 200–1000 bp DNA fragments before they were subjected to immunoprecipitation with primary antibodies against TFAP2C (Proteintech, Chicago, IL, USA) or negative control IgG at 4 °C overnight. Finally, the purified DNA was detected by qRT–PCR using the P1-P7 primer sequences listed in Additional file 1: Table S3.

### Immunohistochemistry (IHC)

The collected subcutaneous tumors were fixed with 4% formalin, embedded in paraffin and then sectioned into 4-μm-slices. The standard protocol for IHC of the TMAs and subcutaneous tumors using a streptavidin-peroxidase (SP) Kit (Zhongshan biotech, Beijing, China), as described previously [25]. The slides were incubated with antibodies specific for TFAP2C (Abcam, cambridge, MA, USA), TRIM29 (Proteintech, Chicago, IL, USA), PHLPP1 (Proteintech, Chicago, IL, USA), p-AKT (Cell Signaling Technology, Danvers, MA, USA) and Ki-67 (Cell Signaling Technology, Danvers, MA, USA). The images of IHC staining were obtained by an Olympus microscope (Tokyo, Japan).

### Statistical analysis

Statistical analyses were performed using SPSS 19.0 software (IBM, Armonk, NY, USA) and GraphPad Prism version 8.0 (La Jolla, CA, USA). All data in the current study are presented as the means ± standard deviation (SD), and all the tests were two-sided. A *P* value of < 0.05 was considered statistically significant. Student’s t-test or one-way analysis of variance (ANOVA) was used to evaluate the significance of differences between groups. The correlation analysis was detected by Pearson’s correlation coefficient. Receiver operating characteristic (ROC) curves were used to analyze the sensitivity and specificity of circIL4R detection, whereas the relationship of circIL4R expression and the clinicopathological parameters of CRC was calculated by the chi-square test; The Overall survival (OS) and disease-free survival (DFS) were calculated by the Kaplan–Meier method and log-rank test. The univariate and multivariate Cox proportional hazard regression models were used to determine the effects of circIL4R or other clinicopathological parameters on survival and evaluate the hazard ratio.

### Further applied methods

Additional RNase R treatment and actinomycin D, luciferase reporter, fluorescence in situ hybridization (FISH), biotinylated RNA pull-down, western blot and coimmunoprecipitation (Co-IP), Transwell migration and invasion, wound healing, cell counting kit-8 (CCK-8), 5-ethynyl-2′-deoxyuridine (EdU), colony formation assays, assessment of IHC, in situ hybridization (ISH) and animal experiments are described in Additional file 2: Supplemental Materials and Methods.

## Results

### CircIL4R is identified and characterized in CRC cells

**Fig. 1:**
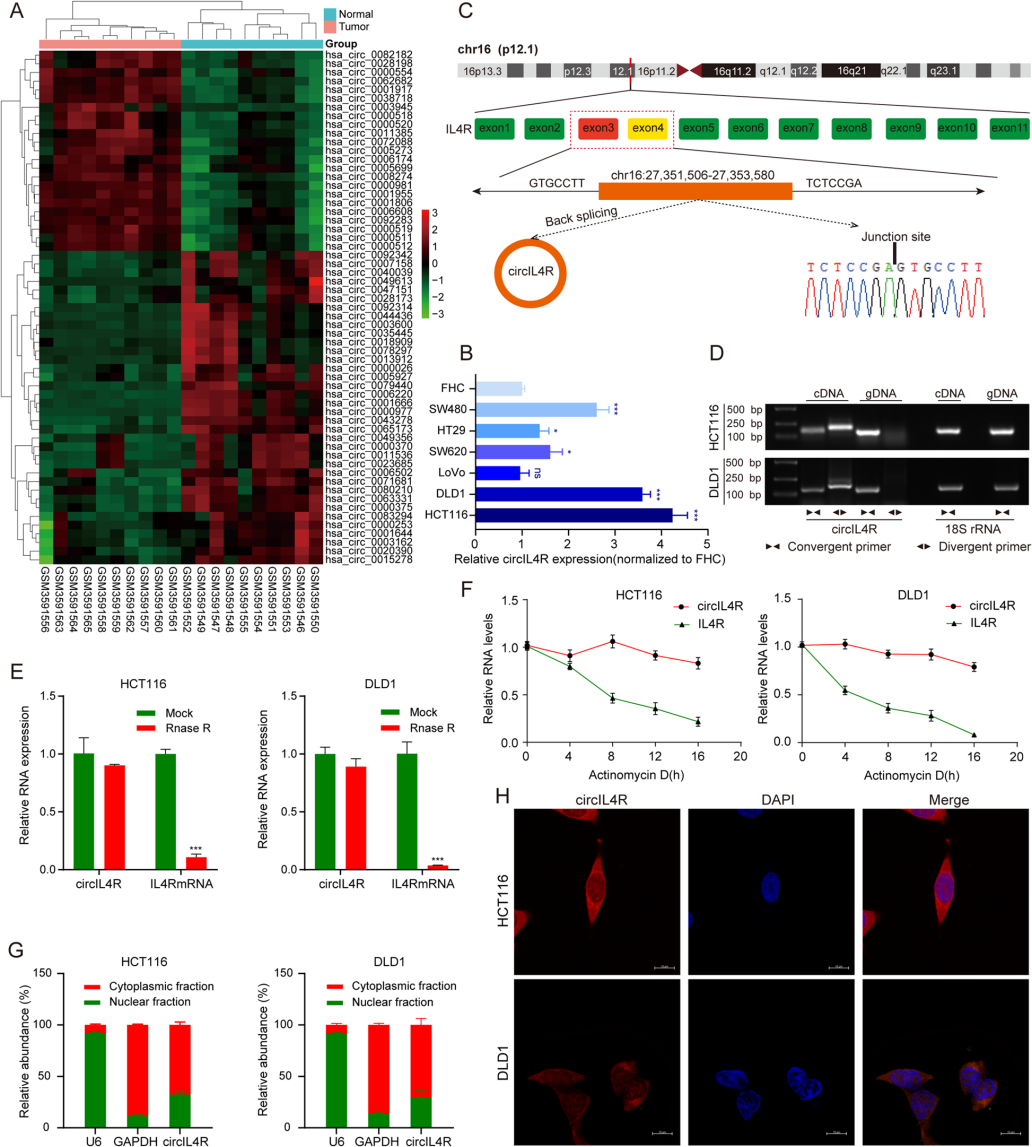
Identification and characterization of circIL4R in CRC cells. **a** Heatmap showing the significantly differentially expressed circRNAs in ten pairs of CRC tissues and corresponding normal tissues (*n* = 20). Red and green denote upregulated and downregulated circRNAs, respectively. **b** Relative expression of circIL4R in a normal colorectal epithelium cell line (FHC) and CRC cell lines (HCT116, DLD1, LoVo, SW620, HT29 and SW480). 18S rRNA served as internal reference. **c** Schematic illustration of the formation of circIL4R via circularization from exons 3 to 4 in the IL4R gene. **d** The existence of circIL4R was examined by agarose gel electrophoresis with the PCR products. circIL4R could be amplified by divergent primers in the complementary DNA (cDNA) but not genomic DNA (gDNA). 18S rRNA was used as a positive control. **e** The expression of circIL4R and IL4R mRNA in CRC cells treated with RNase R was examined by qRT-PCR. **f** The stability of circIL4R and IL4R mRNA in CRC cells treated with actinomycin D at the indicated times was examined by qRT-PCR. **g** and **h** qRT-PCR assay of the subcellular fractionation products and representative FISH images showing that the localization of circIL4R in CRC cells was primarily in the cytoplasm. *P < 0.05, **P < 0.01, ***P < 0.001

To identify novel oncogenic circRNAs that contribute to CRC progression, we conducted bioinformatic analysis based on a publicly available GEO dataset (GSE126094) comprising ten pairs of CRC tissues and ANTs. Overall, based on |fold-change| ≥2 and *P value* < 0.05, the analysis revealed a total of 59 significantly dysregulated circRNAs and is presented in the cluster heatmap; twenty-three circRNAs were upregulated, and thirty-six circRNAs were downregulated (Fig. 1a). To further verify these expression patterns in CRC cell lines, we selected fourteen candidate circRNAs that were upregulated in the GEO database and have not been reported in cancers. In general, the novel circRNA hsa_circ_0038718 exhibited the most significant upregulation in HCT116, DLD1, LoVo, SW620, HT29, and SW480 CRC cells compared to FHC cells; therefore, we focused on this circRNA for further study (Fig. S1a, Fig. 1b and Fig. S3h). Next, the circular structure of hsa_circ_0038718 was investigated based on the annotation of the circBase database. Sequence analysis showed that hsa_circ_0038718 was generated from exons 3 and 4 of the IL4R gene (Chr16: 27,351,506-27,353,580) with a length of 227 nt; thus, we designated it circIL4R in this study (Fig. 1c). To further confirm the circular form of circIL4R in CRC, convergent primers were designed to amplify the linear forms of IL4R and mRNA, while divergent primers were designed to amplify the circular forms of circIL4R. cDNA and gDNA extracted from HCT116 and DLD1 cells were used as templates for PCR. Agarose gel electrophoresis assays showed that circIL4R was amplified only in cDNA but not in gDNA (Fig. 1d). Furthermore, RNase R digestion assays revealed that circIL4R, but not linear IL4R mRNA, was resistant to RNase R treatment (Fig. 1e). Similarly, actinomycin D RNA stability assays showed that circIL4R exhibited a longer half-life than that of the linear IL4R (Fig. 1f). These results demonstrated the existence of a circular form of circIL4R in CRC. Subsequently, nuclear-cytoplasmic fractionation and FISH assays were performed to detect the subcellular localization of circIL4R. The results indicated that circIL4R was mainly localized to the cytoplasm of CRC cells (Fig. 1g, h). Taken together, these results revealed that circIL4R, located in the cytoplasm, was a highly stable and frequently upregulated circRNA in CRC.

### Clinical significance of circIL4R expression in CRC patients

**Fig. 2:**
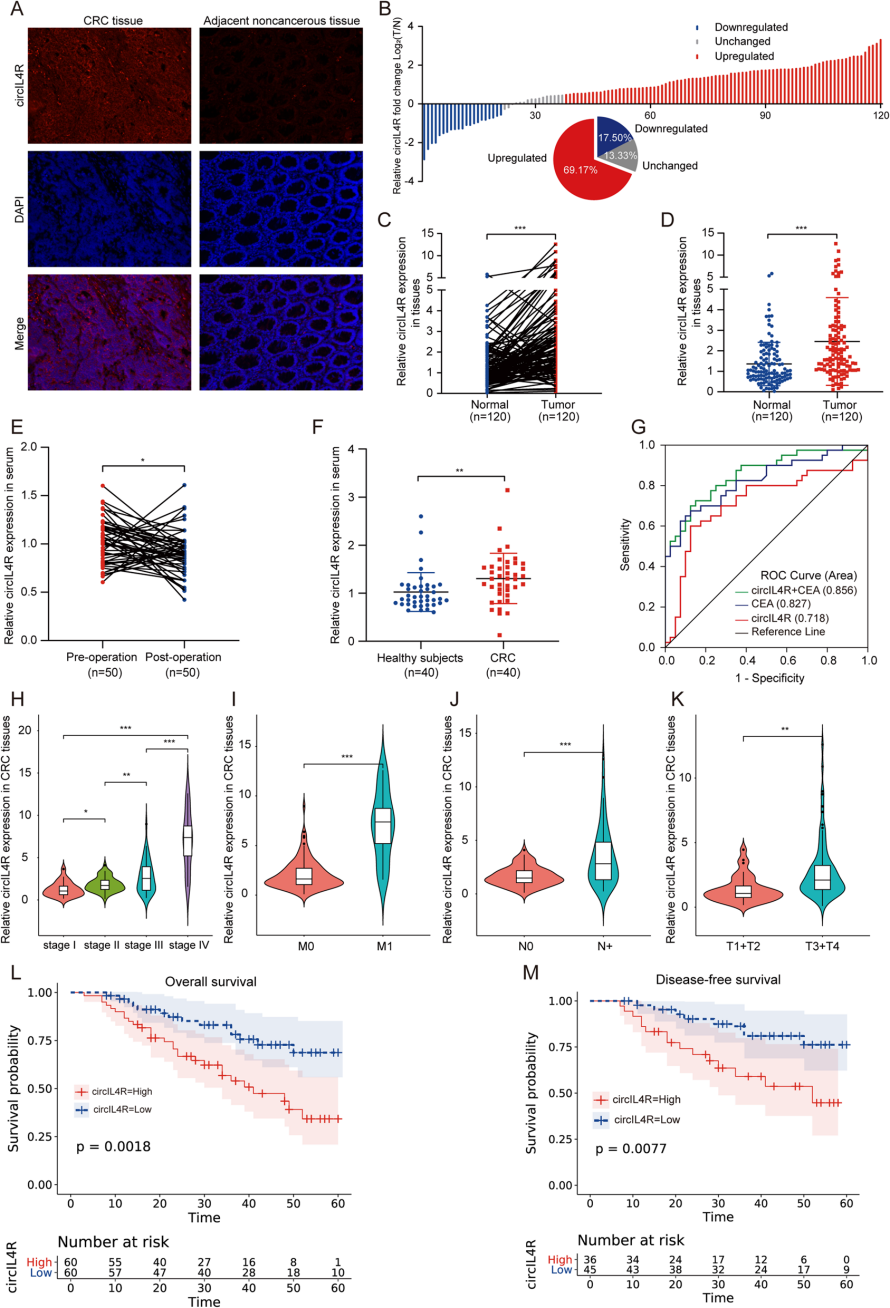
Clinical significance of circIL4R in CRC tissues and serum. **a** FISH staining assay showed that circIL4R expression in CRC tissues was higher than that in the corresponding ANTs (200×). **b-d** qRT-PCR analysis showed that circIL4R expression was significantly upregulated in 120 CRC tissues compared with paired ANTs. **e** Changes in the levels of serum circIL4R in 50 newly diagnosed CRC patients before and after surgery were detected by qRT-PCR. **f** Relative levels of serum circIL4R in healthy controls (*n* = 40) and CRC patients (*n* = 40) were determined by qRT-PCR. **g** ROC curves were used to determine the diagnostic value of serum circIL4R either alone or in combination with CEA in CRC. **h-k** circIL4R expression was evaluated in different groups stratified according to clinical characteristics (pathological stage, M classification, N classification, T classification.) by violin plot. **l** and **m** Kaplan–Meier survival curves showed that CRC patients with high circIL4R expression exhibited shorter OS (*n* = 120, *P* = 0.0018) and DFS (*n* = 120, *P* = 0.0077) based on 120 clinical specimens. *P < 0.05, **P < 0.01, ***P < 0.001

To determine the clinical significance of circIL4R in CRC patients, we first validated its expression in a cohort of 120 CRC patients with follow-up data. The results of the FISH staining and qRT–PCR assays showed that circIL4R was significantly upregulated in CRC tissues compared with ANTs, which was consistent with the bioinformatic analysis of the GEO database (Fig. 2a-d). Given that circRNAs are highly stable and not easily degraded, we were interested in whether circIL4R can be used as a diagnostic and prognostic biomarker for CRC. We first examined the pre- and postoperative serum levels of circIL4R in another cohort of 50 CRC patients. The results indicated that the serum circIL4R was reduced in the postoperative samples compared with the preoperative samples (Fig. 2e). Next, we assessed the serum expression of circIL4R in another cohort of 40 CRC patients and 40 healthy subjects and found that the serum circIL4R was significantly higher in CRC patients than in the healthy subjects (Fig. 2f). Additionally, to assess the value of circIL4R expression as a serum biomarker for distinguishing healthy people and CRC patients, an ROC curve was generated based on the data from our previous cohort (40 CRC patients and 40 healthy subjects), and the area under the ROC curve (AUC) was calculated. The AUC of circIL4R was 0.718 (95% CI = 0.599–0.837); at the cutoff value of 1.184, the Youden index was 0.475, with a sensitivity of 60% and an optimal specificity of 87.5%. This indicates that circIL4R expression may be valuable as a serum biomarker of CRC. To compare the specificity and sensitivity of circIL4R with those of the well-known biomarker CEA, the ROC curve of CEA was calculated, and the AUC was 0.827 (95% CI = 0.736–0.917), which was considerably higher than the AUC value obtained for circIL4R. More importantly, we established a diagnostic panel comprising the detection of circIL4R and CEA, which showed significantly superior diagnostic efficiency, with an AUC of 0.856 (95% CI = 0.773–0.938), indicating the promising value of this biomarker panel for diagnosing CRC (Fig. 2g). As we continued to investigate the expression of circIL4R in the CRC cohort comprising 120 pairs of CRC tissues and ANTs, we were surprised to find that circIL4R expression was different in groups classified by tumor pathological stage, T classification, N classification, and M classification (Fig. 2h-k).

To further analyze the relationship between circIL4R expression and clinicopathological features, a chi-square test was performed, and we found that increased circIL4R expression was positively associated with aggressive features of CRC, such as tumor diameter (*P* = 0.022), depth of invasion (*P* = 0.001), lymph node metastasis (*P* = 0.010), distant metastasis (*P* = 0.038), and TNM stage progression (*P* = 0.003), suggesting that circIL4R plays an oncogenic role to drive the proliferation and metastasis of CRC. Furthermore, there was no clear relationship between circIL4R expression and age, sex, or differentiation state (Additional file 1: Table S4). Subsequently, the prognostic signature of circIL4R in CRC based on the survival follow-up information of 120 patients was analyzed. Kaplan–Meier survival curves showed that circIL4R overexpression was positively associated with poor OS (*P* = 0.0018) and DFS (*P* = 0.0077) (Fig. 2l, m). Univariate Cox regression analysis suggested that circIL4R expression, depth of invasion, lymph node metastasis (LNM), differentiation, TNM stage, and distant metastasis were significant risk factors for OS, while circIL4R expression, depth of invasion, LNM, differentiation, tumor diameter and TNM stage were significant risk factors for DFS (Additional file 1: Table S5). The multivariate analysis showed that circIL4R expression could serve as an independent prognostic biomarker for OS (HR, 2.077; 95% CI, 1.079–3.998; *P* = 0.029) and DFS (HR, 2.927; 95% CI, 1.179–7.270; *P* = 0.021) in CRC patients (Additional file 1: Table S6). Additionally, circIL4R expression was detected by ISH in the TMAs comprising 179 pairs of CRC tissues and ANTs. The results of ISH revealed that circIL4R expression in CRC tissues was much higher than that in ANTs, and further analysis showed that circIL4R expression was remarkably higher in CRC tissues of stage III/IV than those of stage I/II (Fig. S4a-d). In addition, there was a positive association between circIL4R expression and tumor diameter, depth of invasion, lymph node metastasis, distant metastasis, TNM stage and poor prognosis (Fig. S4e, Additional file 1: Table S8). In conclusion, these results indicated that circIL4R may serve as an effective prognostic marker and novel therapeutic target for CRC.

### TFAP2C induces circIL4R expression via transcriptional regulation in CRC

**Fig. 3:**
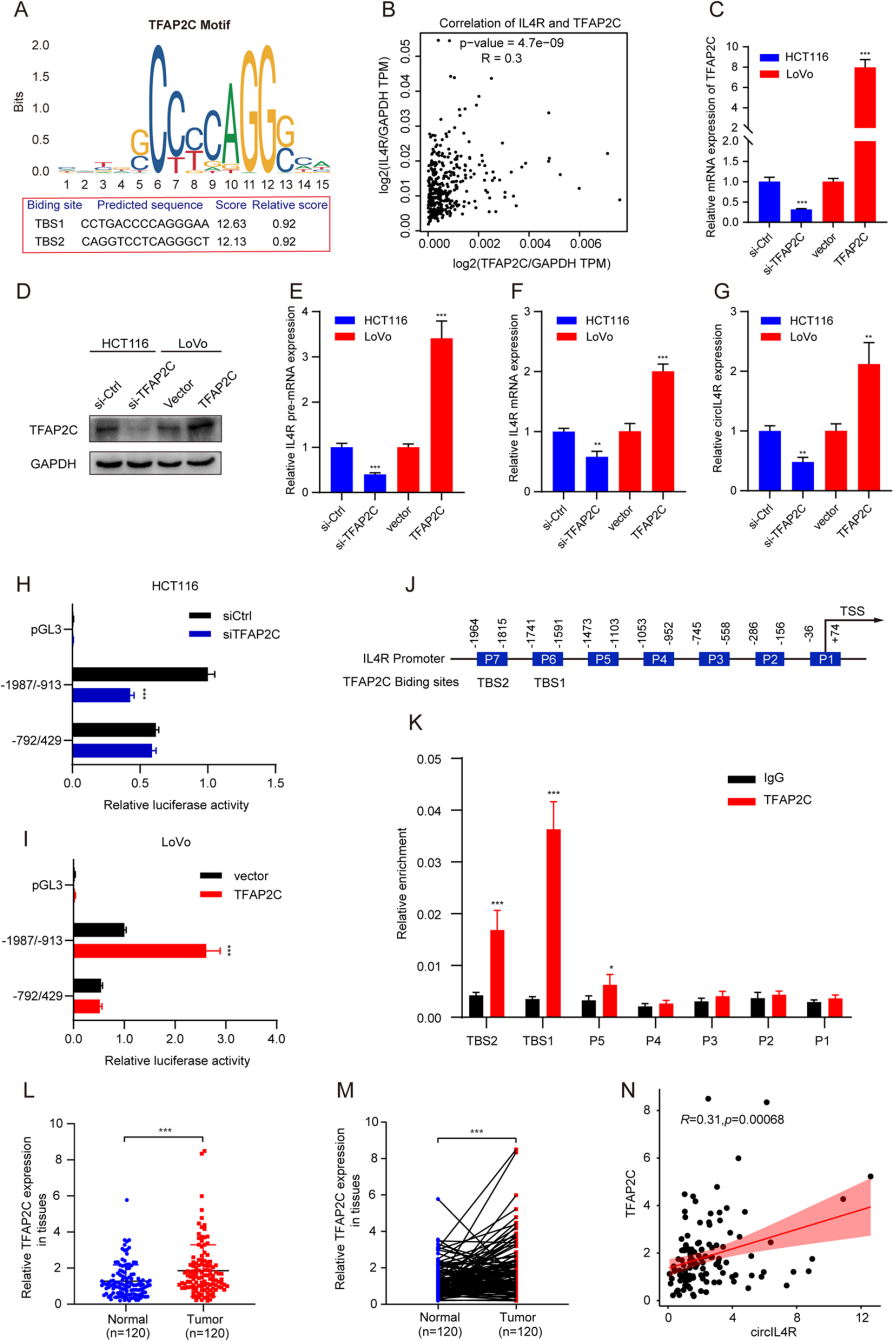
TFAP2C enhances circIL4R expression via transcriptional regulation. **a** The predicted TFAP2C-binding sequence in the IL4R promoter region was identified with the JASPAR website; the scores of the predicted sequences termed TBS1 and TBS2 are shown. **b.** TFAP2C expression in CRC was positively correlated with IL4R expression based on the analysis of the GEPIA online dataset. **c** and **d** qRT-PCR and western blot assays validating the TFAP2C mRNA and protein expression in HCT116 and LoVo cells transfected with siRNAs and overexpression plasmids, respectively. **e-g** qRT-PCR showed that the expression of pre-mRNA, mRNA and circRNA of IL4R was significantly upregulated in CRC cells transfected with TFAP2C overexpression plasmids but downregulated in CRC cells with TFAP2C knockdown. **h** and **i** Luciferase reporter assays of the pGL-IL4R promoter showed that luciferase activity driven by the − 1987/− 913 fragment of the IL4R promoter region was significantly reduced in HCT116 cells with TFAP2C knockdown but enhanced in LoVo cells with TFAP2C overexpression. **j** Schematic illustration of the seven IL4R promoter regions (named P1–P7) designed for TFAP2C potential binding regions (*above*). Schematic illustration of predicted TFAP2C binding sites (named TBS1 and TBS2) (*below*). **k** The ChIP assay and qRT-PCR analysis showed that TFAP2C bound to the TBS1-TBS2 and P5 regions in the IL4R promoter; IgG was used as the negative control. **l** and **m** qRT-PCR analysis showed that TFAP2C mRNA expression was significantly upregulated in 120 CRC tissues compared with that in paired ANTs. **n**. The mRNA expression of TFAP2C in CRC was positively correlated with circIL4R based on the qRT-PCR results. *P < 0.05, **P < 0.01, ***P < 0.001

We further explored the potential mechanism driving circIL4R upregulation in CRC. Previous studies have reported that transcription factors (TFs) can enhance the expression of circRNAs by modulating the transcription of their host genes [26, 27]; therefore, we used the JASPAR database and performed BLAST on the TFs that could recognize the sequences in the IL4R promoter region. Among the predicted TFs, we focused on TFAP2C because of its two high affinity response elements located within the IL4R promoter region, which were named TBS1 and TBS2 (Fig. 3a). In addition, TFAP2C was shown to be upregulated and to exhibit oncogenic activity in CRC [28]. Moreover, the correlation analysis provided by the GEPIA database indicated that TFAP2C was positively associated with IL4R (Fig. 3b). Next, TFAP2C overexpression plasmids or siRNAs were constructed and transfected, and the relative expression of TFAP2C was validated in CRC cells by qRT–PCR and western blot (Fig. 3c, d). To explore the effects of TFAP2C on the expression of circIL4R in CRC, we designed primers for IL4R pre-mRNA, mRNA and circRNA. The qRT–PCR results showed that the expression of pre-mRNA, mRNA and circRNA of IL4R was significantly upregulated in CRC cells transfected with TFAP2C overexpression plasmids, but downregulated upon TFAP2C knockdown (Fig. 3e-g). Based on this finding, we speculated that TFAP2C activate the transcription of IL4R pre-mRNA, which may lead to upregulation of IL4R mRNA and circIL4R. To further test this hypothesis, three different fragments of the IL4R promoter were cloned into luciferase reporter vectors. The results of luciferase reporter assays indicated that the luciferase activity driven by the − 1987/− 913 fragment was significantly reduced in cells with TFAP2C knockdown (Fig. 3h) but enhanced in cells with TFAP2C overexpression (Fig. 3i). Nevertheless, changes in TFAP2C expression had no effect on the luciferase activity driven by the − 792/429 fragment (Fig. 3h, i). These results confirmed that TFAP2C-responsive sites were contained within the − 1987/− 913 region of the IL4R promoter. Next, seven pairs of primers (denoted P1–7) were designed for different regions of the IL4R promoter (Fig. 3j). The ChIP and quantitative PCR results showed that TFAP2C bound to regions P5–7. Notably, P5 and P6 were also referred to as TFAP2C binding site 1 (TBS1) and TBS2 in the IL4R promoter (Fig. 3k). In addition, we validated the mRNA expression of TFAP2C by qRT–PCR in a cohort of 120 CRC patients, and detected the protein level of TFAP2C by IHC in the TMAs. The results showed that TFAP2C expression was significantly upregulated in CRC tissues compared with ANTs and exhibited a positive association with circIL4R (Fig. 3l-n, Fig. S4f, g, j).

### CircIL4R promotes CRC cell proliferation, migration and invasion in vitro

**Fig. 4:**
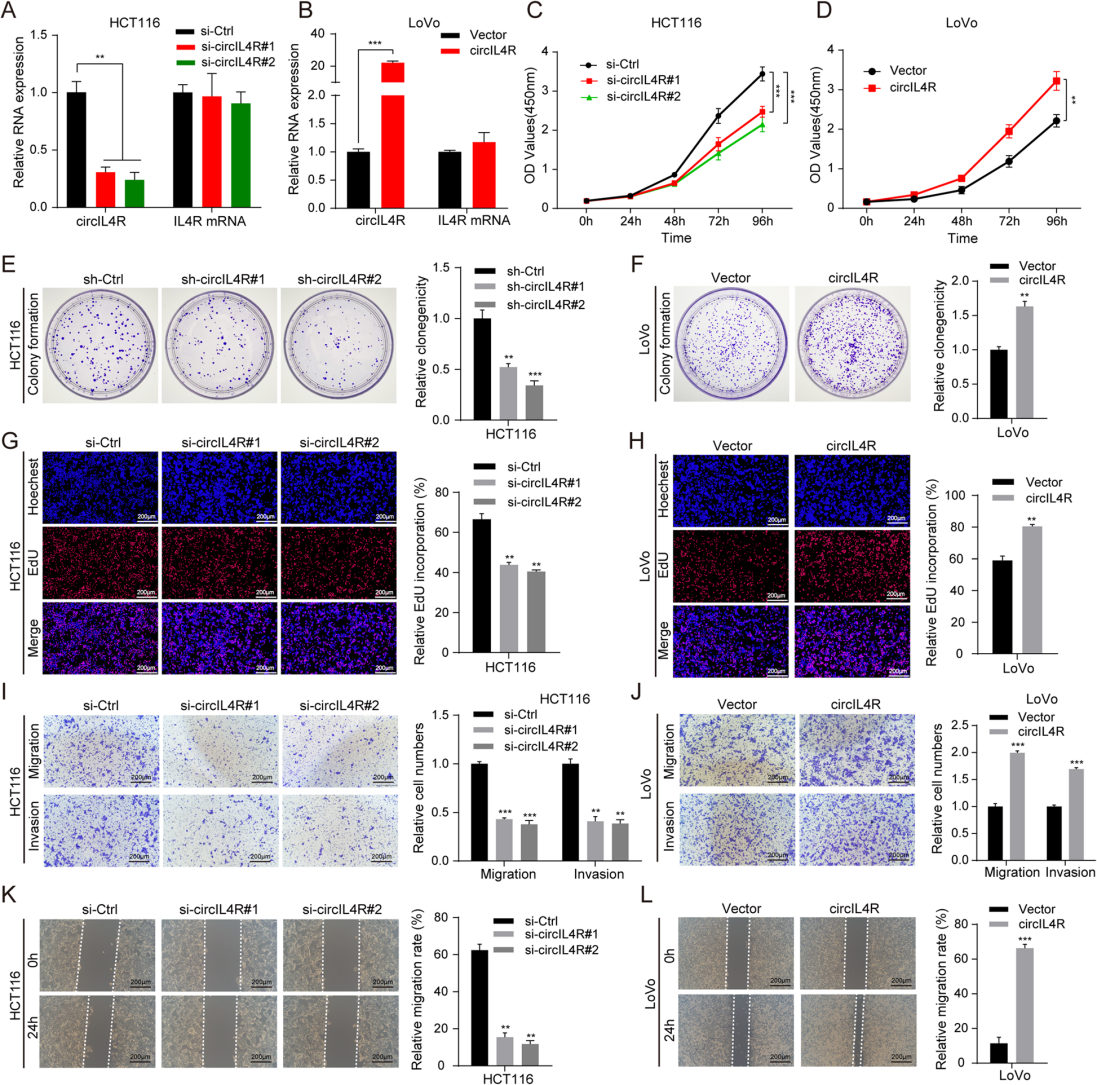
circIL4R facilitates the proliferation, migration and invasion of CRC cells in vitro*.* **a** and **b** qRT-PCR validation of circIL4R and IL4R mRNA expression in HCT116 and LoVo cells transfected with siRNAs and overexpression plasmids, respectively. **c** and **d** The viability curves of CRC cells with knockdown or overexpression of circIL4R were detected by the CCK-8 assay at the indicated time points. **e** and **f** A colony formation assay was conducted to determine the proliferation of CRC cells stably transfected with sh-Ctrl, sh- circIL4R, vector or circIL4R. **g** and **h** An EdU assay was performed to assess the proliferation ability of CRC cells transfected with the indicated siRNAs or plasmids. **i **to** l** Representative images and quantification of Transwell and wound healing assays of CRC cells transfected with the indicated siRNAs or plasmids. The data are presented as the mean ± SD. *P < 0.05, **P < 0.01, ***P < 0.001

To investigate the biological function of circIL4R in CRC cells, we constructed two siRNAs (si-circIL4R#1 and si-circIL4R#2) targeting circIL4R and one overexpression plasmid of circIL4R and performed qRT–PCR assays to detect circIL4R expression in different CRC lines (HCT116, DLD1, LoVo, SW620, HT29, and SW480) and a normal colorectal epithelial cell line (FHC). The results of Fig. 1b show that circIL4R expression is significantly upregulated in HCT116 and DLD1 cells and relatively low in LoVo cells. Hence, we chose HCT116 and DLD1 cells for circIL4R knockdown via transfection with siRNAs against circIL4R, while LoVo cells were chosen for overexpression of circIL4R via transfection with circIL4R overexpression plasmids; neither transfection had any influence on the level of IL4R mRNA (Fig. 4a, b and Fig. S1b). Subsequently, HCT116 and DLD1 cell lines with stable circIL4R knockdown (sh-circIL4R#1 and sh-circIL4R#2) or a negative control vector were constructed by lentiviral transfection. CCK-8, EdU and colony formation assays demonstrated that circIL4R knockdown markedly suppressed the proliferation of CRC cells, whereas circIL4R overexpression exerted the opposite effects (Fig. 4c-h and Fig. S1c-e). These results clearly suggested that circIL4R promotes the proliferation of CRC cells.

Given that circIL4R expression was positively associated with lymph node metastasis, distant metastasis and depth of invasion in CRC patients, we wanted to determine whether circIL4R facilitates migration and invasion in CRC cells. Transwell and wound healing assays were performed to detect the effects of circIL4R on the metastasis of CRC cells. The results revealed that downregulation of circIL4R expression remarkably suppressed the migratory and invasive abilities of HCT116 and DLD1 cells. Conversely, overexpression of circIL4R significantly enhanced these abilities in LoVo cells (Fig. 4i-l and Fig. S1f, g). Collectively, these findings demonstrated that circIL4R promotes CRC cell proliferation, migration and invasion in vitro.

### CircIL4R promotes aggressive behaviors of CRC cells via the PI3K/AKT signaling pathway

**Fig. 5:**
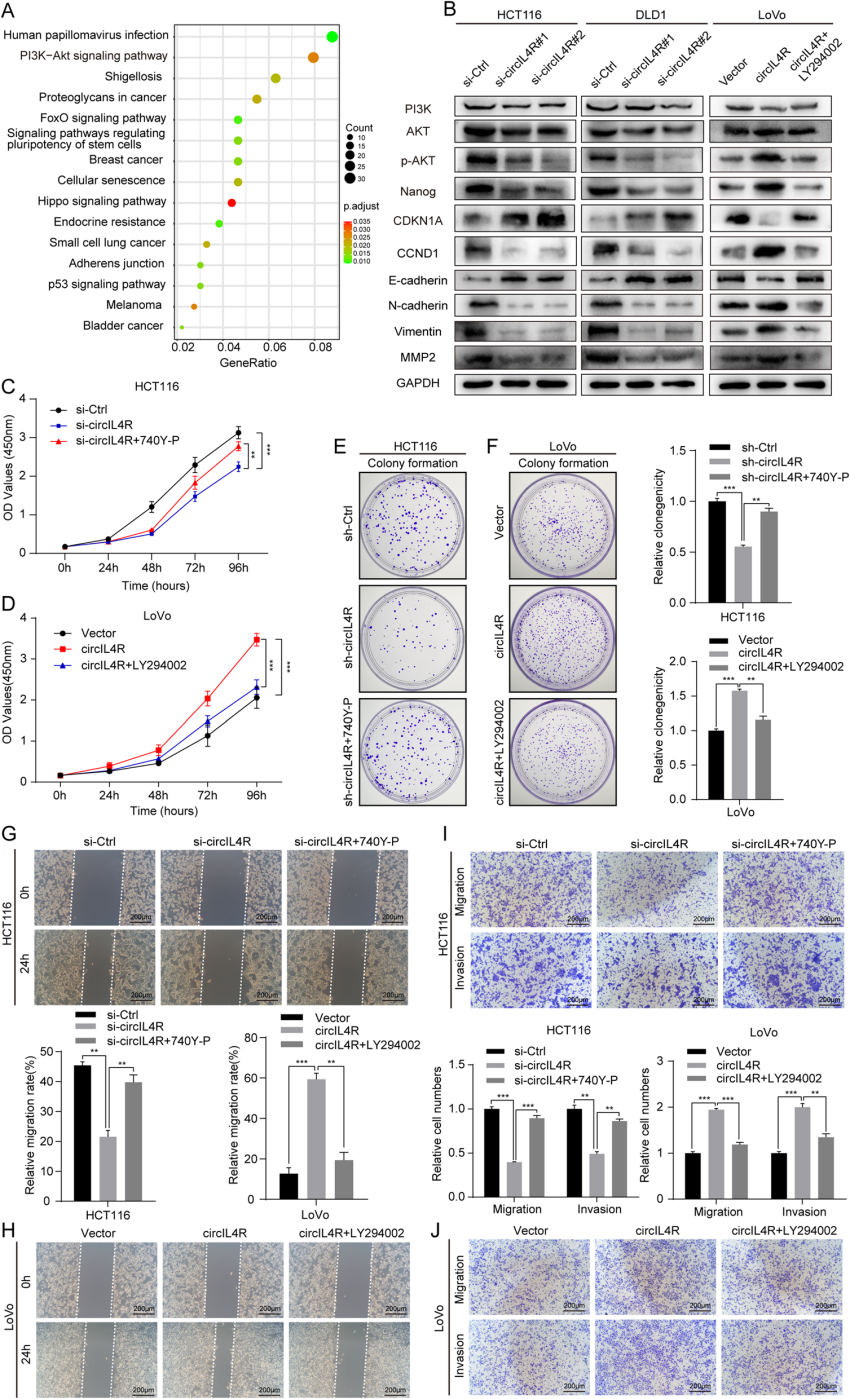
circIL4R activates the PI3K/AKT signaling pathway. **a** Identification of circIL4R related signaling pathways by KEGG analysis. **b** The expression of constituents of the PI3K/AKT signaling pathway and its corresponding downstream genes in CRC cells with knockdown or overexpression of circIL4R, and the effect of LY294002 on PI3K/AKT pathway were determined by western blot. **c-f** The CCK-8 and colony formation assays indicated that the inhibitory effect of circIL4R knockdown on the proliferation ability of HCT116 cells was reversed by treatment with 740Y-P, an activator of the PI3K/AKT signaling pathway, whereas the stimulatory effect of circIL4R overexpression on the proliferation ability of LoVo cells was reversed by LY294002, an inhibitor of the PI3K/AKT signaling pathway. **g**-**j** Representative images and quantification of Transwell and wound healing assays showed that the inhibitory effect of circIL4R knockdown on the migration and invasion ability of HCT116 cells was reversed by 740Y-P, whereas the stimulative effect of circIL4R overexpression on the migration and invasion ability of LoVo cells was reversed by LY294002. The data are presented as the means ± SD. *P < 0.05, **P < 0.01, ***P < 0.001

To further explore the potential molecular mechanism and signaling pathways underlying the effect of circIL4R on CRC progression, bioinformatic analysis was carried out based on the target genes of circIL4R. The KEGG analysis results revealed that circIL4R was remarkably related to the PI3K-Akt signaling pathway (Fig. 5a), which has been reported to play an important role in CRC cell proliferation and metastasis [29–31]; thus, we inferred that circIL4R promotes CRC progression through the PI3K/AKT signaling pathway. To confirm this hypothesis, western blot analysis was performed. The results showed that knockdown of circIL4R in HCT116 and DLD1 cells significantly reduced the expression levels of p-AKT and its related downstream genes, such as Nanog, CyclinD1 (CCND1), N-cadherin, Vimentin and MMP2, and increased the expression of p21 (CDKN1A) and E-cadherin; the total protein levels of PI3K and AKT seemed stable (Fig. 5b). These findings suggested that circIL4R activates the PI3K/AKT signaling pathway in CRC cells. To further clarify whether the PI3K/AKT pathway mediates the regulatory effect of circIL4R on the aggressive behaviors of CRC cells, 740Y-P, an activator of the PI3K/AKT signaling pathway, and LY294002, an inhibitor of the PI3K/AKT signaling pathway, were used to perform rescue experiments in cells with circIL4R knockdown and overexpression, respectively. Next, the results of western blot assay showed that the effect of circIL4R overexpression in LoVo cells on protein levels of PI3K/AKT related genes were partially reversed using the PI3K selective inhibitor LY294002 (Fig. 5b). In addition, the inhibitory effect of circIL4R knockdown on the proliferation, migration and invasion ability of HCT116 and DLD1 cells was clearly mitigated in the presence of 740Y-P, whereas the stimulatory effect of circIL4R overexpression on the proliferation, migration and invasion ability of LoVo cells was clearly diminished in the presence of LY294002 (Fig. 5c-j). Taken together, these findings indicated that circIL4R promotes the proliferation, migration and invasion of CRC cells via activation of the PI3K/AKT signaling pathway.

### CircIL4R serves as a sponge for miR-761 in CRC cells

**Fig. 6:**
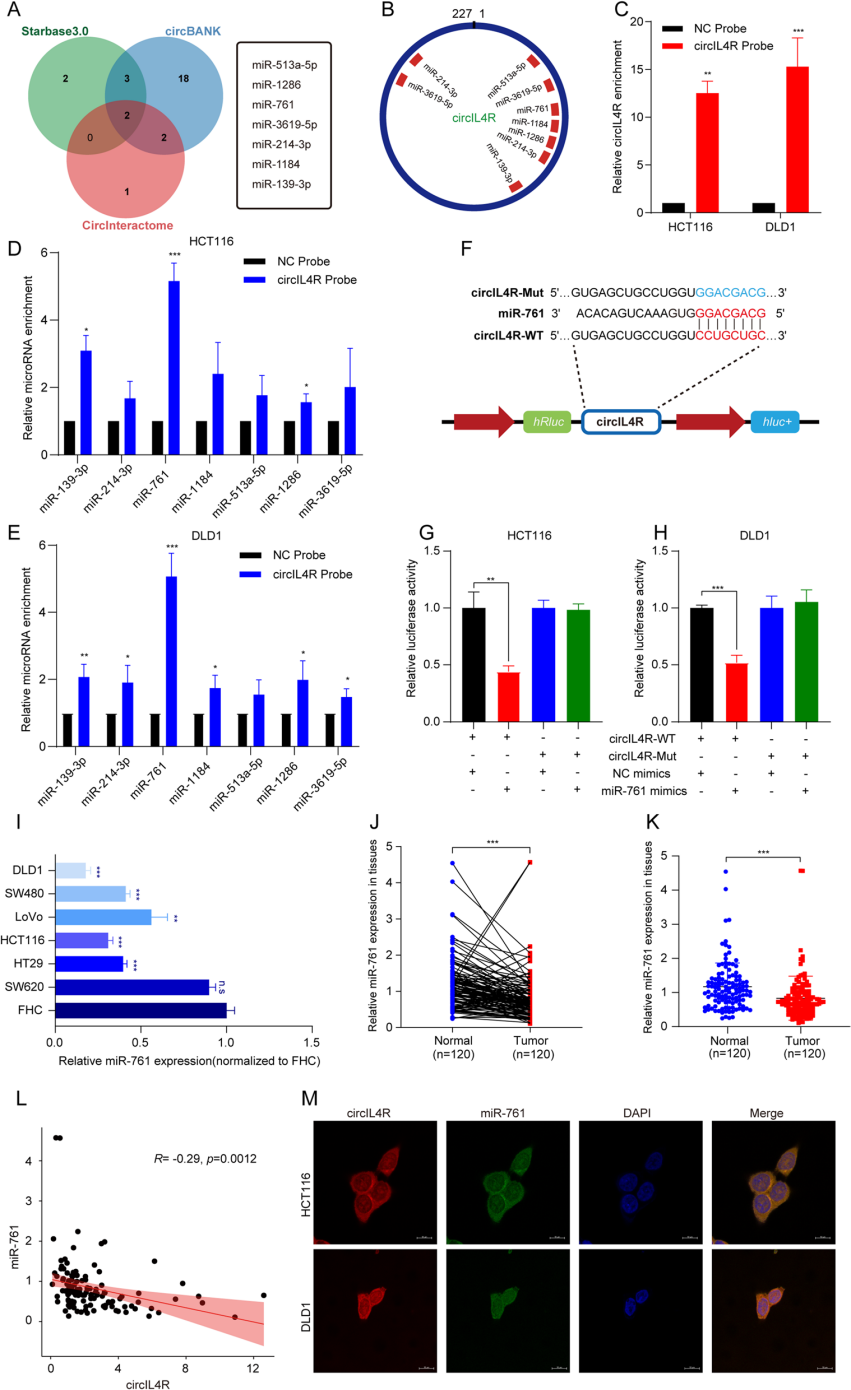
circIL4R acts as a sponge for miR-761 in CRC cells. **a** Seven miRNAs potentially targeted by circIL4R were predicted by cross-analysis with Circular RNA Interactome, circBANK and starbase3.0 databases. **b** Schematic illustration of the putative binding sites of seven miRNA candidates predicted to bind with circIL4R. **c** The efficiency of the biotinylated circIL4R probe in CRC cells was validated by qRT-PCR. **d** and **e** The relative expression of the miRNA candidates enriched by the circIL4R probe in CRC cells was assessed by qRT-PCR. **f** Schematic illustration of WT and Mut circIL4R luciferase reporter vectors. **g** and **h** CRC cells were cotransfected with miR-761 mimics and either WT or Mut circIL4R luciferase reporter vectors, and the activity of the luciferase reporter was determined. **i** Relative expression of miR-761 in a normal colorectal epithelium cell line (FHC) and CRC cell lines (HCT116, DLD1, LoVo, SW620, HT29 and SW480). **j-l** qRT-PCR analysis showed that miR-761 expression was significantly downregulated in 120 CRC tissues compared with paired ANTs and was negatively correlated with circIL4R expression. **m** Colocalization between circIL4R and miR-761 in CRC cells was verified by FISH staining. DAPI was used to stain the nuclei. Scale bar, 10 μm. *P < 0.05, **P < 0.01, ***P < 0.001

We next explored the specific mechanism by which circIL4R affects CRC progression. Accumulating evidence has confirmed that cytoplasmic circRNAs usually function as miRNA sponges to regulate the expression of downstream target gene [12]. Since circIL4R was predominantly located in the cytoplasm of CRC cells, and a previous study reported that circIL4R can function as a sponge for miR-541-3p in hepatocellular carcinoma (HCC) cells [32]. However, our results demonstrated that circIL4R could not function via miR-541-3p in CRC cells (Fig. S3i, j). To further explore the binding partners of circIL4R in CRC cells, we first conducted a cross-analysis with three online miRNA target prediction databases: Circular RNA Interactome, circBANK and Starbase3.0. In total, seven miRNAs (miR-513a-5p, miR-1286, miR-761, miR-3619-5p, miR-214-3p, miR-1184, miR-139-3p) emerged as potential targets for circIL4R across two of the three databases (Fig. 6a). Furthermore, the putative binding sites of circIL4R are shown in Fig. 6b. Next, RNA pull-down assays were performed to further confirm the binding partners of circIL4R, and the efficiency of the biotin-labeled circIL4R probe was verified by qRT–PCR (Fig. 6c). The results showed that miR-761 was the most highly enriched miRNA by the circIL4R probe in HCT116 and DLD1 cell lines (Fig. 6d, e). To clarify the interaction between circIL4R and miR-761, we constructed a luciferase reporter containing wild-type (WT) or mutant (Mut) sequences, which were designed based on the putative binding sites for circIL4R and miR-761 (Fig. 6f). The luciferase reporter assay demonstrated that transfection of miR-761 mimics significantly suppressed the luciferase activities in cells expressing the circIL4R WT reporter, while no significant difference was detected in cell expressing the circIL4R Mut reporter group (Fig. 6g, h). Then, we explored the expression of miR-761 in a cohort of 120 CRC patients and CRC cell lines. The qRT-PCR results showed that in contrast to circIL4R, miR-761 was remarkably downregulated in CRC tissues relative to paired ANTs and shared a negative correlation with circIL4R (Fig. 6j-l). Additionally, the expression of miR-761 was significantly downregulated in CRC cell lines relative to that in FHC cells (Fig. 6i). More importantly, the results of the double FISH assay showed the colocalization of circIL4R and miR-761 in the cytoplasm of CRC cells (Fig. 6m). Collectively, our data indicated that circIL4R may function as a sponge for miR-761 in CRC cells.

Previous studies have reported that miR-761 can attenuate the malignant progression of CRC and deactivate the PI3K/Akt signaling pathway in osteosarcoma [33, 34]. Thus, to further elucidate whether circIL4R activates the PI3K/AKT pathway by interacting with miR-761, rescue experiments were performed by cotransfecting miR-761 mimics or inhibitor with the circIL4R overexpression plasmid or corresponding siRNA. First, qRT–PCR assays were performed to verify the efficiency and specificity of the miR-761 mimics and inhibitor in CRC cells (Fig. S2a-c). The results of the functional assays showed that transfection of miR-761 mimics remarkably attenuated the malignant effects of proliferation, migration and invasion caused by circIL4R overexpression in LoVo cells, while transfection of miR-761 inhibitor rescued the suppressive effects of circIL4R knockdown in HCT116 and DLD1 cells (Fig. S2d-i). Western blot analysis showed that the reduced levels of p-AKT in HCT116 cells with circIL4R knockdown could be rescued by cotransfection of miR-761 inhibitor. Similarly, the opposite effects could be observed in LoVo cells (Fig. S2j, k). Taken together, these findings demonstrated that circIL4R promotes proliferation, migration, and invasion and activates the PI3K/AKT pathway in CRC cells by interacting with miR-761.

### TRIM29 is a downstream target of miR-761

**Fig. 7:**
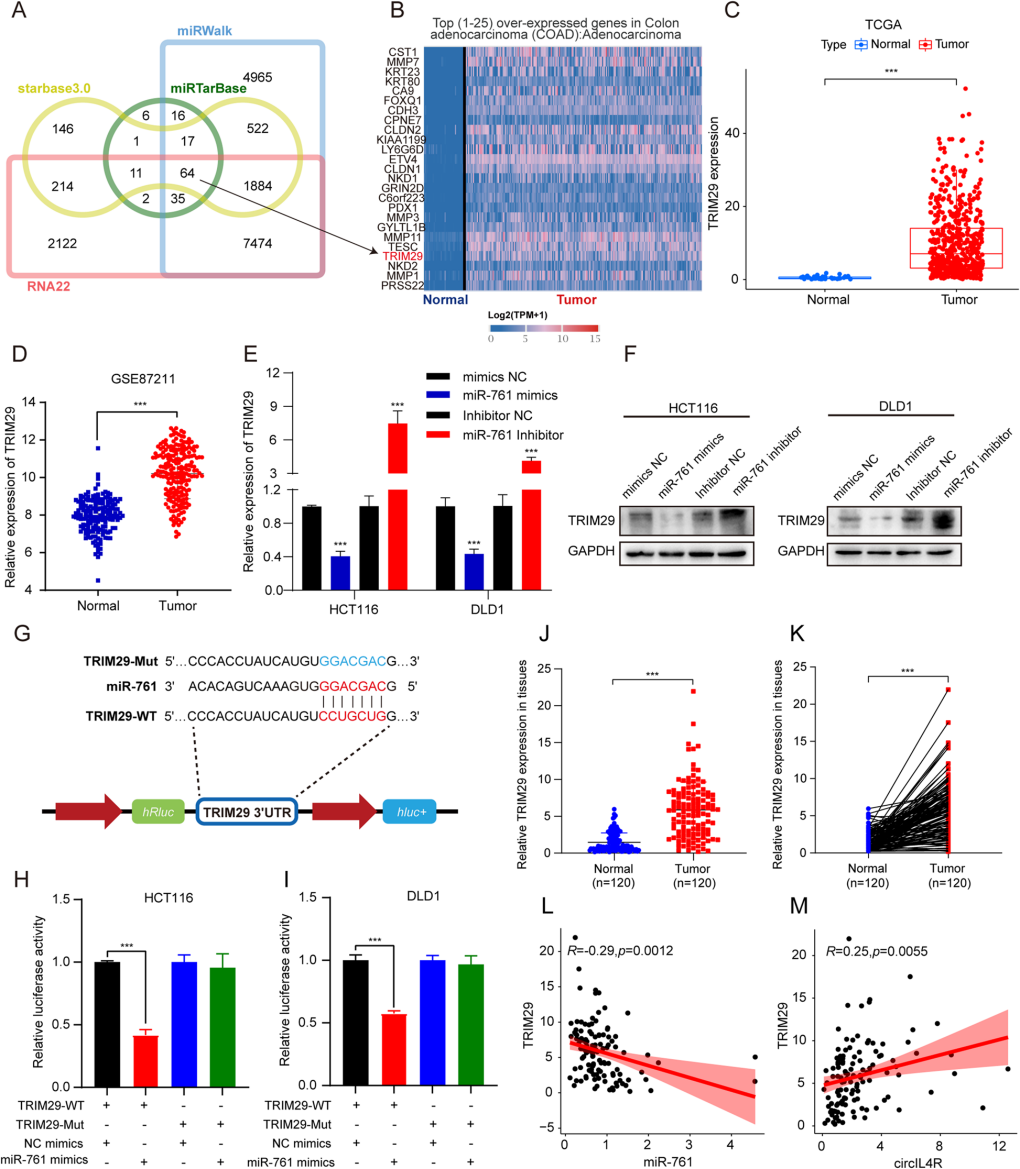
TRIM29 is a downstream target of miR-761. **a** Overlapping of the target genes of miR-761 predicted by cross-analysis of starbase3.0, RNA22, miRWalk and miRTarBase. **b** Heatmap of top 25 overexpressed genes in the TCGA-COAD database. **c** TRIM29 expression at the mRNA level in CRC tumors and corresponding normal tissues from the TCGA database. **d** TRIM29 expression in the primary tumors and normal tissues of CRC patients from the GEO database. **e** and **f** The mRNA and protein levels of TRIM29 were assessed by qRT-PCR and western blot in CRC cells transfected with miR-761 mimics or inhibitor. **g** Schematic illustration of WT and Mut TRIM29 luciferase reporter vectors. **h** and **i** CRC cells were cotransfected with miR-761 mimics and either WT or Mut TRIM29 luciferase reporter vectors, and the activity of the luciferase reporter was determined. **j** and **k** qRT-PCR analysis showed that TRIM29 expression was significantly upregulated in 120 CRC tissues compared with the paired ANTs**. l** and **m** TRIM29 expression in CRC was positively correlated with circIL4R and negatively correlated with miR-761 based on qRT-PCR. *P < 0.05, **P < 0.01, ***P < 0.001

To systematically identify biologically relevant miR-761 target genes in CRC, we performed bioinformatic analysis using online databases Starbase3.0, RNA22, miRWalk and miRTarBase. Cross-analysis based on the four databases revealed 64 candidate genes targeted by miR-761 (Fig. 7a, Additional file 1: Table S7). Next, we assessed the expression of these candidate genes in colon and rectal cancer tissues. Among the predicted target genes of miR-761, TRIM29 became our primary focus because of its remarkable upregulation in CRC samples compared to normal tissues, and its inclusion in the heatmap of the top 25 overexpressed genes in the TCGA database (Fig. 7b, c and Fig. S3a). Similar to the TCGA findings, TRIM29 expression was also upregulated in the database from GEO database (GSE87211) containing 273 CRC tissues and 160 normal tissues (Fig. 7d). Subsequently, the results of the qRT–PCR assays and western blot analysis showed that the transfection of miR-761 mimics significantly reduced the TRIM29 expression at both the mRNA and protein levels in CRC cells, while transfection of miR-761 inhibitor elicited the opposite results (Fig. 7e, f). To further elucidate the interaction between miR-761 and TRIM29, we constructed luciferase reporters containing a WT or Mut TRIM29 3′-UTR, which were designed based on the putative binding sites for miR-761 and TRIM29 (Fig. 7g). The luciferase reporter assay demonstrated that transfection of miR-761 mimics significantly suppressed the luciferase activities of cells expressing the TRIM29 WT reporter, while no significant difference was detected in cells expressing the TRIM29 Mut reporter (Fig. 7h, i), indicating that miR-761 reduced TRIM29 expression by directly binding to the 3′-UTR of TRIM29. Clinically relevant results showed, the mRNA expression of TRIM29 was remarkably upregulated in a cohort of 120 CRC tissues and displayed a negative correlation with the level of miR-761 and a positive correlation with the level of circIL4R (Fig. 7j-m). Moreover, results of IHC staining showed that the TRIM29 expression was upregulated in CRC tissues compared with ANTs, and shared a positive correlation with circIL4R expression in TMAs (Fig. S4h, i, k). Collectively, these findings indicated that TRIM29 is a direct target of miR-761.

### CircIL4R facilitates CRC progression and activates the PI3K/AKT signaling pathway via the miR-761/TRIM29 axis

**Fig. 8:**
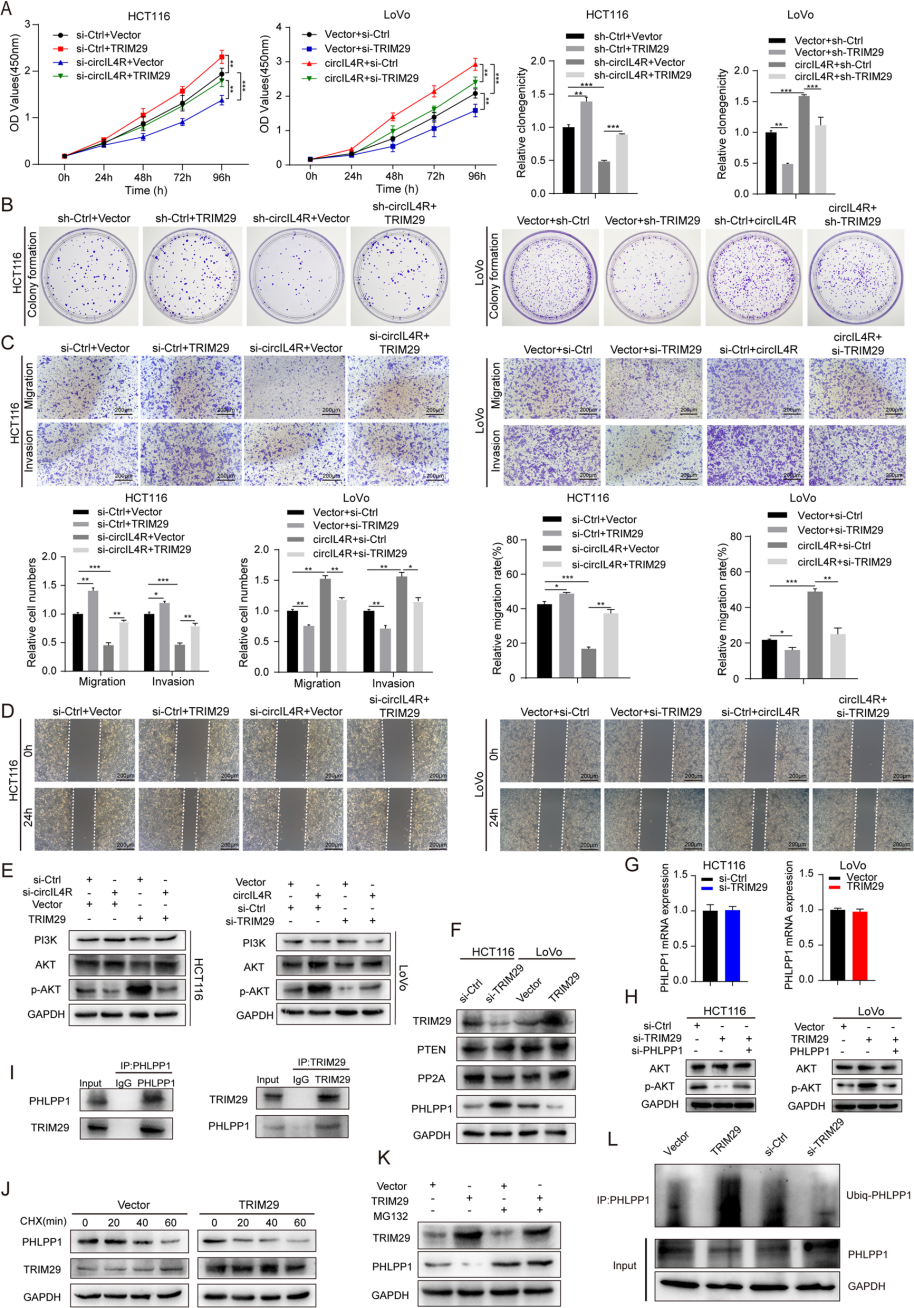
circIL4R facilitates CRC progression through the circIL4R/miR-761/TRIM29/PHLPP1 axis. **a** and **b** CCK-8 and colony formation assays indicated that the inhibitory effect of circIL4R knockdown on the proliferation of HCT116 cells was reversed by TRIM29 overexpression, whereas the stimulatory effect of circIL4R overexpression on the proliferation of LoVo cells was reversed by TRIM29 knockdown. **c** and **d** Representative images and quantification of Transwell and wound healing assays showed that the inhibitory effect of circIL4R knockdown on the migration and invasion of HCT116 cells was reversed by TRIM29 overexpression, whereas the stimulatory effect of circIL4R overexpression on the migration and invasion of LoVo cells was reversed by TRIM29 knockdown. **e** Western blots showed that the reduction in p-AKT in HCT116 cells transfected with circIL4R siRNAs was reversed by TRIM29 overexpression, whereas the increase in p-AKT in LoVo cells transfected with circIL4R was reversed by TRIM29 knockdown. **f** The protein levels of PTEN, PP2A and PHLPP1 in CRC cells transfected with TRIM29 siRNAs or overexpression plasmids were detected by western blot. **g** The mRNA level of PHLPP1 in CRC cells transfected with TRIM29 siRNAs or overexpression plasmids was detected by qRT-PCR, respectively. **h** The protein level of p-AKT in HCT116 cells transfected with siTRIM29, either alone or with siPHLPP1 (*left*) and in LoVo cells transfected with the TRIM29 overexpression plasmid, either alone or with the PHLPP1 overexpression plasmid (*right*). **i** The interaction between TRIM29 and PHLPP1 in HCT116 cells was detected by immunoprecipitation and western blot assays. **j** The effect of TRIM29 overexpression on the half-life of PHLPP1 protein in HCT116 cells was detected by western blot. The cells were treated with 100 μg/ml CHX, a protein synthesis inhibitor, and harvested at the indicated time points. **k** HCT116 cells were treated with MG132 (10 μM), and harvested at the indicated times to measure total protein levels via western blot assays. The results showed that TRIM29 overexpression decreased the protein level of PHLPP1 in a proteasome-dependent manner. **l** The effect of TRIM29 overexpression or knockdown on the ubiquitination of PHLPP1 in HCT116 cells was assessed by western blot. *P < 0.05, **P < 0.01, ***P < 0.001

As a downstream target of miR-761, TRIM29 has been confirmed to be an oncogene in CRC and can promote proliferation, migration, invasion and glucose metabolism in CRC [22, 23]. Of clinical significance, TRIM29 exhibited a close correlation with circIL4R and miR-761. Therefore, we further investigated whether circIL4R and TRIM29 are functionally associated. First, an siRNA targeting TRIM29 (si-TRIM29) and a TRIM29 overexpression plasmid were transfected into CRC cells. The transfection efficiency was verified at the protein and mRNA levels by western blotting and qRT–PCR assays, respectively (Fig. S3b-c). Subsequently, the results of the CCK-8, colony formation, Transwell and wound healing assays verified that TRIM29 overexpression promoted the proliferation, migration and invasion of CRC cells, whereas TRIM29 knockdown exhibited the opposite effects (Fig. 8a-d). More importantly, we observed that the inhibited proliferation, migration and invasion abilities of HCT116 cells caused by circIL4R knockdown could be hindered by TRIM29 overexpression, while the malignant progression of LoVo cells resulting from circIL4R overexpression could be largely blocked by TRIM29 knockdown (Fig. 8a-d). We next investigated whether circIL4R activates the PI3K/AKT signaling pathway through the miR-761/TRIM29 axis. Western blot assays revealed that decreased p-AKT levels caused by circIL4R knockdown in HCT116 cells could be reversed by TRIM29 overexpression in HCT116 cells (Fig. 8e). Conversely, upregulation of p-AKT levels induced by circIL4R overexpression could be blocked by TRIM29 knockdown (Fig. 8e). Taken together, these findings demonstrated that the role of circIL4R in promoting the activation of the PI3K/AKT signaling pathway and the regulation of CRC malignant progression was largely dependent on the miR-761/TRIM29 axis.

### CircIL4R activates the PI3K/AKT signaling pathway via TRIM29-mediated ubiquitination degradation of PHLPP1

As circIL4R can enhance the expression of TRIM29 expression by sponging miR-761, forced overexpression of TRIM29 could activate the PI3K/AKT signaling pathway. Thus, we elucidated the molecular mechanism by which circIL4R activates PI3K/AKT via the miR-761/TRIM29 axis. A previous study discovered that in nasopharyngeal carcinoma, TRIM29 overexpression can inhibit the expression of PTEN expression and activate the PI3K/AKT pathway [35]. It is known that the tumor suppressors PTEN, PP2A, and PHLPP1 can inhibit the activity of the PI3K/AKT pathway [36–38]. To determine whether TRIM29 can regulate the PI3K/AKT signaling pathway in the same manner, a western blot assay was performed, the results of which revealed that neither PTEN nor PP2A expression was changed by knockdown or overexpression of TRIM29 (Fig. 8f). However, TRIM29 knockdown upregulated the protein expression of PHLPP1, while overexpression of TRIM29 exhibited the opposite effect; there were no significant effect on the mRNA level of PHLPP1 (Fig. 8f, g). Moreover, western blot analysis showed that TRIM29 knockdown could depress p-AKT levels. Cotransfection of si-TRIM29 and si-PHLPP1 reversed this effect, and vice versa (Fig. 8h). These findings indicated that in CRC, TRIM29 may activate the PI3K/AKT signaling pathway via posttranscriptional modification of PHLPP1. Previous publications have reported that PHLPP1 activity could be modulated by multiple posttranslational modifications, including ubiquitination [39]. As an E3 ubiquitin ligase, TRIM29 can facilitate the progression of multiple solid tumors by targeting STING and ISG15 for degradation [19, 40]. Importantly, as a member of the same family as TRIM29, TRIM11 has been reported to degrade PHLPP1 via ubiquitination to activate the PI3K/AKT pathway [41]. We speculated that TRIM29 could also activate the PI3K/AKT pathway by modifying PHLPP1 through ubiquitination. To confirm this hypothesis, we first conducted a coimmunoprecipitation assay with an anti-TRIM29 and an anti-PHLPP1 antibody to detect whether TRIM29 interacts with PHLPP1. The results verified the interaction between endogenous TRIM29 and PHLPP1 proteins in HCT116 cells (Fig. 8i). Next, we examined whether the PHLPP1 protein could be ubiquitinated by TRIM29 in vitro. Cycloheximide (CHX) was used to inhibit protein biosynthesis, and the protein levels at the indicated time points were analyzed by western blot. The results revealed that the half-life of PHLPP1 in HCT116 cells with TRIM29 overexpression was significantly decreased (Fig. 8j), suggesting that TRIM29 could mediate the protein degradation of PHLPP1 protein. Moreover, the regulation of PHLPP1 by TRIM29 could be blocked by the proteasome inhibitor MG132 (Fig. 8k), which further indicated that its regulation may result from the ubiquitination process. More importantly, ubiquitination experiments revealed that that PHLPP1 ubiquitination was reduced in HCT116 cells with TRIM29 knockdown, while TRIM29 overexpression led to the opposite effect (Fig. 8l). Collectively, these findings demonstrated that circIL4R may activate the PI3K/AKT signaling pathway via TRIM29-mediated ubiquitination and consequent degradation of PHLPP1.

### CircIL4R promotes CRC cell proliferation and metastasis in vivo

**Fig. 9:**
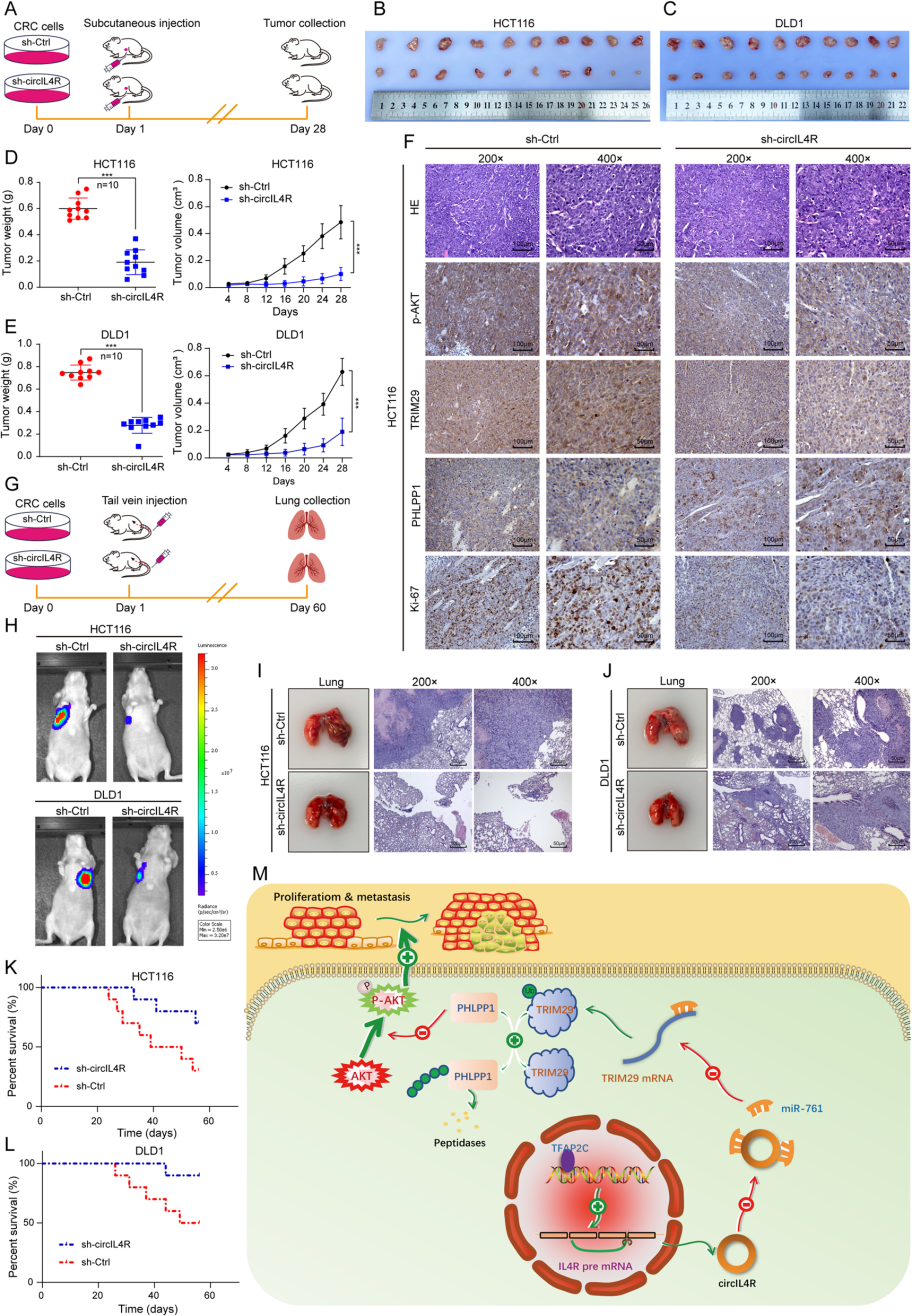
circIL4R promotes the tumorigenesis and metastasis of CRC cells in vivo*.* **a** Schematic illustration of the subcutaneous xenograft tumor model in BALB/c nude mice. **b** and **c** Images of dissected subcutaneous xenograft tumors from different groups. **d** and **e** Growth curve and weight analysis of xenograft tumors in nude mice. **f** Sections of xenograft tumors were subjected to H&E and IHC staining using Ki67, TRIM29, PHLPP1, and p-AKT antibodies. **g.** Schematic illustration of the tail vein-injected nude mouse model. **h** Representative images and luminescence intensity of lung metastatic nodules in tail vein-injected nude mice. (*n* = 5 per group). **i** and **j** Representative images and HE staining of dissected lungs and metastatic nodules. **k** and **l** The survival curves of nude mice injected with CRC cells were determined by the Kaplan–Meier method. **m** Schematic illustration of the mechanism by which circIL4R activates the PI3K/AKT signaling pathway via the miR-761/TRIM29/PHLPP1 axis and promotes proliferation and metastasis in CRC. *P < 0.05, **P < 0.01, ***P < 0.001

To further verify the effect of circIL4R on CRC cell proliferation in vivo, HCT116 and DLD1 cells stably transduced with circIL4R knockdown (sh-circIL4R) or negative control (sh-Ctrl) lentivirus were validated before they were subcutaneously injected into BALB/c nude mice (Fig. S3d, e and Fig. 9a). The results revealed that circIL4R knockdown remarkably inhibited the growth of tumors derived from either CRC cell lines (Fig. 9b, c). Moreover, the volume and weight of the sh-circIL4R-derived tumors were significantly reduced compared to those of the sh-Ctrl group-derived tumors (Fig. 9d, e). In addition, IHC staining revealed that circIL4R knockdown decreased the expression levels of Ki-67, TRIM29 and p-AKT and increased the expression of PHLPP1 (Fig. 9f). Furthermore, luciferase-labeled CRC cells were injected into the tail vein of nude mice to determine whether circIL4R could facilitate the metastasis of CRC cells in vivo (Fig. 9g)*.* The findings revealed that circIL4R knockdown significantly reduced the fluorescence intensity and number of metastatic nodules in the lungs of mice (Fig. 9h-j and Fig. S3f, g). In addition, Kaplan–Meier survival curves showed that the nude mice in the circIL4R knockdown group had longer overall survival than did those in the negative control group (Fig. 9k, l). These findings were consistent with the in vitro experiments, confirming that circIL4R could promote malignant progression of CRC in vivo*.*

## Discussion

In the past few decades, although there have been advances in the clinical treatment of CRC, its prognosis remains unsatisfactory. Therefore, identifying more reliable therapeutic targets and clarifying their effect on CRC progression are urgently needed. Recently, an increasing number of studies have investigated the biogenesis, expression, characteristics, function, and clinical significance of circRNAs in different cancers and emphasized their vital roles in regulating cancer progression [42]. Thanks to the widespread use and availability of high-throughput sequencing and circRNA-specific bioinformatics algorithms, identification and expression data for circRNAs have been uploaded to publicly available databases, which provides opportunities for bioinformatics discovery and clinical validation. In the current study, we identified and verified a novel circRNA termed circIL4R, which was frequently upregulated in microarray analyses, CRC cell lines, and clinical samples and closely associated with malignant clinicopathological factors and an unfavorable prognosis of CRC. The detection of noninvasive biomarkers in fluids is low-cost, can be repeated and has exhibited significant clinical significance in tumor patient management [43]. Due to their high abundance, tissue-specific expression and unique covalent circular bonded structure, circRNAs are attractive candidates for cancer liquid biopsy [44]. In our findings, circIL4R expression was significantly different in the serum of CRC patients before and after surgical resection, as well as of healthy subjects and CRC patients. Notably, the serum expression of circIL4R exhibited a high sensitivity and specificity for CRC diagnosis and surgical efficacy monitoring, which indicated that circIL4R could serve as a liquid diagnostic biomarker. However, we must acknowledge the difficulties and challenges in introducing circRNAs to standard clinical applications. For instance, due to the presence of a back-spliced junction (BSJ) site, specific primers and probes for circRNAs are limited; thus, more sensitive and specific methods are required to increase the accuracy in the detecting of circRNAs [6]. In addition, the identification and detection of circRNA biomarkers in most studies are only preliminary explorations in a relatively small size of sample size, and lagger clinical cohorts and prospective cohorts are required to validate the sensitivity and specificity of circRNAs in diagnosing specific diseases and predicting the prognosis of patients [45]. Thus, optimization of the detection method, validation of the clinical significance and determination of cutoff values would be helpful for incorporating circRNAs as liquid biopsy biomarkers. Additionally, the combined detection of circRNAs and traditional markers such as CEA may be considered an effective approach. Collectively, although many obstacles need to be overcome in the future, the potential of circIL4R as a biomarker for large-scale population screening of CRC and real-time monitoring of CRC progression and treatment effects bring us promising options for liquid biopsy.

Recent studies consistently report that circRNAs can be upregulated in cancers and are correlated with tumorigenesis. Nevertheless, the upstream mechanisms of circRNA overexpression in cancers have rarely been explored. Meng et al. reported that Twist can enhance the expression of circ10720 by binding to the promoter of its host gene [27]. Herein, our data revealed that TFAP2C mediates circIL4R overexpression via transcriptional regulation of its host gene. Our findings are consistent with previous studies reporting that TFAP2C can regulate the expression of lncRNAs by transcriptional activation [46, 47]. Alternatively, previous studies have stated that some RBPs, such as EIF4A3 and FUS, can enhance the generation of circRNAs by binding to their flanking intron sequences [48, 49]. Thus, whether any RBPs participate in the formation, splicing, transport or degradation of circIL4R needs to be further explored.

Our clinical analysis demonstrated that circIL4R overexpression was closely associated with tumor diameter, lymph node metastasis and distant metastasis in CRC patients, suggesting that circIL4R contributes to the proliferation and metastasis of CRC. Subsequently, in vitro gain- and loss-of-function experiments emphasized the significant roles of circIL4R in facilitating CRC cell proliferation, migration and invasion. More importantly, our subcutaneous injection model showed that circIL4R knockdown significantly reduced the tumor burden in vivo, while tail vein injection model revealed that the penetrance of metastasis to distal organs in vivo was dramatically reduced after circIL4R knockdown, including reductions in both the number and size of metastatic nodules. These in vivo and in vitro results revealed that shRNAs or siRNAs specifically targeting circIL4R may effectively inhibit CRC growth and metastasis. In general, these findings demonstrated that circIL4R may serve as an appealing therapeutic target for CRC via RNAi-based strategies. Therefore, the mechanism investigation of circIL4R driving CRC progression was urgently needed. Next, further bioinformatics analysis and validation experiments revealed that circIL4R facilitated the malignant progression of CRC via activation of the PI3K/AKT signaling pathway, which has been reported to participate in a wide range of biological processes, including the regulation of CRC progression [31]. Xu et al. found that lncRNA MALAT1 activates the PI3K/AKT signaling pathway via the miR-26a/26b/FUT4 axis and promotes proliferation and metastasis in CRC [50]. Kumar S et al. demonstrated that IDO1 promotes CRC cell proliferation and inhibits apoptosis via activation of the PI3K/AKT signaling pathway [51]. Nevertheless, there is little evidence about the relationship between circRNAs and the PI3K/AKT signaling pathway in CRC.

Next, we focused on the mechanism by which circIL4R activates the PI3K/AKT signaling pathway and promotes CRC proliferation and metastasis. Since the intracellular localization of circRNA is critical to its regulatory mechanism and function, our FISH and nuclear-cytoplasmic fractionation results revealed that circIL4R is mainly distributed in the cytoplasm. Accumulating evidence shows that circRNAs located in the cytoplasm can regulate biological functions by effectively sponging miRNAs to regulate the expression of their target genes [52]. Our previous study indicated that circSPARC overexpression upregulates the JAK2 expression by sponging miR-485-3p, thereby activating the JAK2/STAT3 pathway and enhancing CRC progression [53]. In the current study, we observed that circIL4R can act as a sponge to competitively bind with miR-761, as verified by RNA pull-down, dual-luciferase reporter, and FISH assays. miR-761 was previously reported to be a tumor suppressor that was downregulated in several cancers and to deactivate the PI3K/AKT signaling pathway [33, 34, 54, 55]. In agreement with previous reports, our data revealed that miR-761 expression could inhibit the proliferation and metastasis of CRC cells and was downregulated in CRC tissues and cells. Subsequent rescue experiments further revealed that miR-761 reversed the oncogenic roles of circIL4R and its ability to activate the PI3K/AKT signaling pathway in CRC. In addition to functioning as competitive endogenous RNAs, circRNAs can encode cancer-related peptides or bind with specific protein(s) to implement their functions [11, 56]. To explore whether circIL4R could activate the PI3K/AKT signaling pathway and facilitate CRC progression by encoding protein/peptides or binding with specific protein(s), we first searched the circRNADb online database and found no open reading frame (ORF) in the sequence of circIL4R, indicating that no protein features were predicted and that the possibility of encoding protein by circIL4R was relatively low. Additionally, the cat RAPID online database predicted that circIL4R has the potential to bind with several proteins, such as SRSF9 and PTBP1. Previous studies have reported that these two proteins were involved in alternative splicing [57, 58]. Whether circIL4R could regulate the alternative splicing via binding with SRSF9 or PTBP1 need further investigation.

Next, upon further investigation of the underlying downstream mechanism of miR-761, TRIM29 was identified as the target of miR-761 based on the cross-analysis of four microRNA targeted gene prediction databases and the publicly available TCGA and GEO databases. Notably, the interaction between miR-761 and TRIM29 has been described in previous studies. Guo et al. indicated that miR-761 facilitated the malignant phenotypes of triple-negative breast cancer cells by targeting TRIM29 [59]. Herein, we confirmed the interaction between miR-761 and TRIM29 in CRC by qRT–PCR, western blotting and dual-luciferase reporter assays. More importantly, the results of rescue experiments further demonstrated that TRIM29 overexpression reversed the inhibition of functional phenotypes and deactivation of the PI3K/AKT signaling pathway caused by circIL4R knockdown, suggesting that the circIL4R/miR-761/TRIM29 axis facilitated the proliferation and metastasis of CRC cells by regulating the PI3K/AKT signaling pathway.

Nevertheless, to date, the mechanism by which the circIL4R /miR-761/TRIM29 axis contributes to PI3K/AKT signaling pathway activation remains elusive. Therefore, further investigations to elucidate the mechanism by which TRIM29 activates the PI3K/AKT signaling pathway are necessary. As a member of the tripartite motif protein family, TRIM29 has been shown to play oncogenic roles in various cancers, including CRC, and activate the PI3K/AKT signaling pathway in thyroid cancer [22–24]. To date, whether and how forced overexpression of TRIM29 contributes to the activation of the PI3K/AKT signaling pathway in CRC remains unclear. However, the tumor suppressor genes PTEN, PHLPP1, and PP2A all inhibit the activity of the PI3K/AKT pathway [60]. Zhou et al. reported that TRIM29 overexpression can inhibit PTEN expression and activate the PI3K/AKT pathway in nasopharyngeal carcinoma [35]. Phillip L et al. found that TRIM29 could also induce the epigenetic silencing of PTEN in bladder cancer [20]. Therefore, we speculated that TRIM29 may activate the PI3K/AKT pathway by regulating PTEN in CRC. Contrary to our expectations, there were no significant changes in PTEN expression when TRIM29 the expression was altered. We speculated that the reason for this phenomenon may be the specificity of cancer cells types. Intriguingly, the protein level of PHLPP1 was significantly upregulated upon TRIM29 knockdown, while the mRNA level was not changed. Moreover, the results of the rescue experiments further revealed that the deactivation of the PI3K/AKT pathway caused by TRIM29 knockdown could be reversed upon PHLPP1 knockdown. These findings suggested that TRIM29 activates the PI3K/AKT signaling pathway via posttranscriptional modification of PHLPP1. A previous study reported that the E3 ubiquitin ligase TRIM11, also a member of the tripartite motif protein family, could degrade PHLPP1 via ubiquitination to activate the PI3K/AKT pathway [41]. We therefore examined whether TRIM29 interacted with PHLPP1. Subsequently, a series of experiments revealed that TRIM29 knockdown was accompanied by reduced levels of ubiquitinated PHLPP1, while TRIM29 overexpression was accompanied by enhanced levels of ubiquitinated PHLPP1. Therefore, we surmised that TRIM29 targets PHLPP1 for ubiquitin-mediated degradation, thereby resulting in PI3K/AKT signaling pathway activation. However, future studies are needed to fully investigate the domain required for PHLPP degradation.

## Conclusions

In summary, our findings provide the first evidence that circIL4R has significantly increased expression in CRC cells, tissues, and serum and can function as a diagnostic and prognostic biomarker. More importantly, we found that TFAP2C induced circIL4R upregulation via transcriptional regulation and that circIL4R overexpression competitively interacted with miR-761 to enhance TRIM29 expression, thereby targeting PHLPP1 for ubiquitin-mediated degradation to activate the PI3K/AKT signaling pathway and facilitate CRC progression, as shown in Fig. 9m and Fig. S3k. Further research on circIL4R may provide novel insights into CRC diagnosis and treatment, as well as significantly advance therapies in clinical practice.

## Supplementary Information

**Additional file 1: Table S1.** Clinical characteristics of 120 CRC samples used for qRT-PCR validation. **Table S2.** The sequences of siRNAs and shRNAs used in this study. **Table S3.** The sequences of primers used for qRT-PCR. **Table S4.** Relationship between circIL4R expression and clinicopathological features in fresh-frozen specimens. **Table S5.** Univariate Cox regression analysis of circIL4R expression and clinicopathologic variables predicting the survival of CRC patients. **Table S6.** Multivariate Cox regression analysis of circIL4R expression and clinicopathologic variables predicting the survival of CRC patients. **Table S7.** Prediction of miR-761 target genes based on four databases. **Table S8.** Relationship between circIL4R expression and clinicopathological features in TMAs specimens.

**Additional file 2.** Supplemental Materials and Methods.

**Additional file 3: Figure S1. a.** qRT-PCR validation of novel circRNAs expressed in different CRC cell lines and FHC cells; the circRNAs are clustered in a heatmap. **b.** qRT-PCR validation of circIL4R and IL4R mRNA expression in DLD1 cells transfected with siRNAs into DLD1 cells. **c.** The viability of DLD1 cells with circIL4R knockdown was detected by CCK-8 assays at the indicated time points. **d.** A colony formation assay was conducted to determine the proliferation of DLD1 cells stably transfected with sh-Ctrl or sh-circIL4R. **e.** An EdU assay was performed to assess the proliferation of DLD1 cells transfected with the indicated siRNAs. **f** and **g.** Representative images and quantification of Transwell and wound healing assays of DLD1 cells transfected with the indicated siRNAs. The data are presented as the means ± SD. *P < 0.05, **P < 0.01, ***P < 0.001. **Figure S2. a-c.** qRT-PCR validation of miR-761 expression in CRC cells transfected with miR-761 mimics or inhibitor into CRC cells. **d-i.** The CCK-8 and Transwell assays showed that the inhibitory effect of circIL4R knockdown on the proliferation, migration and invasion of HCT116 and DLD1 cells was reversed by miR-761 inhibitor, whereas the stimulatory effect of circIL4R overexpression on the proliferation, migration and invasion of LoVo cells was reversed by miR-761 mimics. **j** and **k.** Western blots showed that the reduction in p-AKT levels in HCT116 cells transfected with circIL4R siRNAs was reversed by miR-761 inhibitor, whereas the increase in p-AKT levels in LoVo cells transfected with circIL4R was reversed by miR-761 mimics. *P < 0.05, **P < 0.01, ***P < 0.001. **Figure S3. a.** TRIM29 expression was upregulated in CRC samples based on the TCGA COAD and READ databases. **b** and **c.** The transfection efficiency of the indicated TRIM29 siRNAs or overexpression plasmids was verified at the protein and mRNA level by western blot and qRT-PCR, respectively. **d** and **e.** qRT-PCR validation of circIL4R expression in HCT116 and DLD1 cells stably transfected with circIL4R knockdown (sh-circIL4R) or negative control (sh-Ctrl) lentivirus. **f** and **g.** Analysis of the metastatic nodules from dissected lungs. **h.** Relative expression of circIL4R in a normal colorectal epithelium cell line (FHC) and CRC cell lines (HCT116, DLD1, LoVo, SW620, HT29 and SW480). GAPDH served as internal reference. **i** and **j.** CRC cells were cotransfected with miR-541-3p mimics and either WT or Mut circIL4R luciferase reporter vectors, and the activity of the luciferase reporter was determined. **k.** Schematic illustration of the experimental pipeline by which circIL4R activates the PI3K/AKT signaling pathway via the miR-761/TRIM29/PHLPP1 axis and promotes proliferation and metastasis in CRC. *P < 0.05, **P < 0.01, ***P < 0.001. **Figure S4. a.** Representative images showing the expression of circIL4R in TMAs detected by ISH. **b.** Staining intensities of circIL4R in CRC tissues compared with paired ANTs. N, paired adjacent non-cancerous tissues. C, colorectal cancer tissues (*n* = 165, *P* < 0.001). **c.** The ISH score of circIL4R in CRC tissues was significantly higher than that in paired ANTs. **d.** The ISH score of circIL4R in CRC tissues with stage III/IV was significantly higher than those with stage I/II. **e.** The Kaplan–Meier survival curves showed that CRC patients with high circIL4R expression in the TMAs exerted poor OS. **f.** Representative images showing the expression of TFAP2C in TMAs detected by IHC. **g.** The IHC score of TFAP2C in CRC tissues was significantly higher than that in paired ANTs. **h.** Representative images showing the expression of TRIM29 in TMAs detected by IHC. **i.** The IHC score of TRIM29 in CRC tissues was significantly higher than that in paired ANTs. **j.** Pearson’s correlation analysis of the correlation between circIL4R and TFAP2C expression based on TMAs specimens. **k.** Pearson’s correlation analysis of the correlation between circIL4R and TRIM29 expression based on TMAs specimens. *P < 0.05, **P < 0.01, ***P < 0.001.

## Data Availability

The microarray datasets of dysregulated circRNAs in CRC were obtained from the GEO database (https://www.ncbi.nlm.nih.gov/geo/) under the series accession number GSE126094. The publicly available datasets used in this study can be obtained from their respective online. All datasets used in the present study are available from the corresponding author on reasonable request.

## Abbreviations

CircRNA: Circular RNA
CRC: Colorectal cancer
COAD: Colon adenocarcinoma
READ: Rectal adenocarcinoma
NCCN: National Comprehensive Cancer Network
TCGA: The Cancer Genome Atlas
TRIM29: Tripartite motif-29
cDNA: Complementary DNA
gDNA: Genomic DNA
ROC: Receiver operating characteristic
HE: Hematoxylin eosin
OS: Overall survival
DFS: Disease-free survival
KEGG: Kyoto Encyclopedia of Genes and Genomes
shRNA: Short hairpin RNA
siRNA: Small interfering RNA
GEPIA: Gene Expression Profiling Interactive Analysis
ANTs: Adjacent noncancerous tissues
qRT-PCR: Real-time quantitative polymerase chain reaction
CCK-8: Cell Counting Kit-8
FISH: RNA fluorescence in situ hybridization
IHC: Immunohistochemical staining
ISH: In situ hybridization
EdU: 5-Ethynyl-2′-deoxyuridine
