# Supplementary material for: CircIL4R activates the PI3K/AKT signaling pathway via the miR-761/TRIM29/PHLPP1 axis and promotes proliferation and metastasis in colorectal cancer
Source: Mol Cancer. 2021 Dec 18;20:167. doi: 10.1186/s12943-021-01474-9 (PMC8684286; doi:10.1186/s12943-021-01474-9)
Supplement: Supplementary file 2 — Additional file 2. Supplemental Materials and Methods. [file 12943_2021_1474_MOESM2_ESM.docx]

**Supplemental Materials and Methods**

**RNase R treatment and Actinomycin D assay**

For RNase R treatment assay, total RNA (2ug) of HCT116 and DLD1 cells was incubated with or without 3U/ug RNase R (Epicentre Technologies, Madison, WI, USA) in 37℃ for 30min. Subsequently, the products RNA was purified using RNeasy MinElute Cleanup Kit (Qiagen) and prepared for use. For actinomycin D assay, HCT116 and DLD1 cells were seeded in six-well plates (1×10^5^ cells/well) and cultured for 24 hours. Then, the cells were treated with 2ug/ml actinomycin D (Sigma, USA) and collected at specified time points, respectively. Finally, the RNA stability of IL4R mRNA and circIL4R was detected by qRT-PCR.

**Luciferase reporter assay**

In order to evaluate the direct binding between circIL4R and miR-761, miR-761 and TRIM29, TFAP2C and IL4R promoter region, the related wild-type (WT) or mutant (MUT) response elements was cloned into the pGL3-basic vectors (GeneCreate Biological Engineering Co., Ltd.), which contains the firefly luciferase and renilla luciferase. The WT or MUT of circIL4R and TRIM29 were co-transfected with miR-761 mimics or NC mimics using Lipofactamine 2000 (Invitrogen). Similarly, the truncated IL4R promoter region vectors were co-transfected with TFAP2C overexpression plasmid or siRNAs, respectively. The luciferase activities were detected after 48h by Dual-Luciferase Reporter System Kit (Promega, USA). (Berthold, Germany).

**Fluorescence in situ hybridization (FISH)**

Fluorescein amidite (FAM)-labelled miR-761 probe and Cy3-labelled circIL4R probe were designed and synthesized by RiboBio (Guangzhou, China). The probes were used to detect the location of circIL4R and miR-761 in HCT116 and DLD1 cells by a FISH Kit (RiboBio) according to the manufacturer’s instructions. Besides, 4′,6-diamidino-2-phenylindole, dihydrochloride (DAPI) was used to stain the nuclei of CRC cells. Finally, representative images were taken by a LSM880 NLO confocal microscope system (Carl Zeiss).

**Biotinylated RNA pull-down assay**

In brief, 1×10^7^ HCT116 or DLD1 cells were collected with lysis buffer supplemented with protease inhibitor EDTA-free (Sigma, USA) and RNase inhibitor (Promega). At the same time, Streptavidin-coated magnetic beads (Invitrogen, USA) were incubated with biotinylated-circIL4R or an oligo probe at 25℃ for 2h to construct probe-coated magnetic beads. The cell lysate supernatant was incubated with prepared probe on a rotator at 4℃ overnight. Finally, the RNA complexes bound to the beads were treated with Trizol Reagent (Takara, Japan) to extract RNA for qRT-PCR analysis. The biotinylated circIL4R probe was designed and synthesized by RiboBio (Guangzhou, China), while oligo probe was used as a negative control.

**Western blot and Coimmunoprecipitation (Co-IP) assay**

The transfected CRC cells were harvested and lysed with RIPA buffer (Beyotime, Shanghai, China) supplemented with protease inhibitor cooktail (Thermo Fisher Scientific, USA) and PMSF and quantified by a BCA Protein Assay kit (Beyotime, Shanghai, China). Then, the prepared protein lysates were loaded into a 10% SDS-PAGE gel, transferred onto PVDF membranes (Millipore, Massachusetts, USA). After blocked with 5% skimmed milk, the PVDF membranes were incubated with primary antibodies specific against: TFAP2C (1:1000, Proteintech, Chicago, IL, USA), PI3K (1:1000, Cell Signaling Technology, Danvers, MA, USA), total AKT (1:1000, CST, Danvers, MA, USA), p-AKT (1:1000, CST, Danvers, MA, USA), Nanog (1:1000, CST, Danvers, MA, USA), CDKN1A (1:1000, CST, Danvers, MA, USA), CCND1 (1:1000, CST, Danvers, MA, USA), TRIM29 (1:1000, CST, Danvers, MA, USA), PTEN (1:1000, CST, Danvers, MA, USA), PP2A (1:1000, Proteintech, Chicago, IL, USA), PHLPP1 (1:1500, Proteintech, Chicago, IL, USA), E-cadherin (1:1000, CST, Danvers, MA, USA), N- cadherin (1:1000, CST, Danvers, MA, USA), Vimentin (1:1000, CST, Danvers, MA, USA), MMP2 (1:1000, CST, Danvers, MA, USA) and GAPDH (1:5000, Proteintech, Chicago, IL, USA) at 4℃ overnight. The day after, the PVDF membranes were incubated with corresponding secondary antibody for 2 hours at room temperature before washed with TBST buffer for three times. Finally, Chemistar™ High-sig ECL Western Blot Substrate (Tanon, Shanghai, China) was used to detected the blots. For Co-IP assay, cells were lysed with IP buffer containing protease inhibitors as described above and then incubated with primary TRIM29, PHLPP1 or control IgG antibodies (Beyotime, Shanghai, China) at 4 °C overnight, followed by incubation with protein A/G agarose beads (Santa Cruz Biotechnology, USA) at 4°C for another 4 hours. Subsequently, the bead-bound proteins were washed with lysis buffer for three times and subjected to Western blot analysis with related antibodies against TRIM29, PHLPP1 or Ubiquitin (Abcam, cambridge, MA, USA).

**Transwell migration and invasion assays**

Transwell assay was performed by the chamber with the pore size of 8μm (Corning, NY, USA) according to the manufacturer’s instructions. In invasion assay, upper layer of the chamber plates was treated with Matrigel (BD Biosciences, Mississauga, Canada) while in the migration assay was not. After serum starvation for overnight, transfected CRC cells were seeded in the upper layer of chambers accompanied by complete medium added to the bottom of chambers. After incubation at 37 °C with 5% CO_2_ for 24 to 48 h, the migration and invasion cells were fixed and stained before calculation.

**Wound healing assay**

In wound healing assay, transfected CRC cells were seeded in six-well plates and cultured to the density about 80%. Then, 200-μl pipette tips was used to form the artificial scratches in each well and the exfoliated cells were washed and removed by PBS for twice. The suspended cells were cultured in medium with FBS-free. Inverted light microscope (Olympus, Tokyo, Japan) was used to photograph cell migration distance at 0 h and 24 h.

**Cell Counting Kit-8 (CCK-8) assay**

The CCK-8 assay was performed by Cell Counting Kit 8 (Dojindo Molecular Technologies, Inc., Kumamoto, Japan) according to the manufacturer’s instructions. Transfected CRC cells were seed in 96-well plates and cultured at 37°C in 5 % CO_2_ for 0 h, 24 h, 48 h, 72 and 96h. At specified time‑points, the cells in each well will be treated with 10μL CCK-8 solution and cultured for another 2 hours. Subsequently, the absorbance was measured at 450 nm at the specified time‑points. All the experiments were performed in triplicate.

**5-Ethynyl-2’-deoxyuridine (Edu) assay**

The Edu assay was performed by an Edu Proliferation Kit (RiboBio, Guangzhou, China) according to the manufacturer’s instructions. Transfected CRC cells were seed in 48-well plates and cultured at 37°C in 5 % CO_2_ for 12 h, after which the cells were treated with 50 mM EdU solution at 37°C in 5 % CO_2_ for 2 h and then fixed with 4% paraformaldehyde. Subsequently, the cells were stained with Apollo Dye Solution and Hoechst 33342, the photographed and counted by Olympus FSX100 microscope (Olympus, Tokyo, Japan). These experiments were performed in triplicate.

**Colony formation assay**

Stably transfected CRC cells were seed in six-well plants (800 cells/well) and cultured in corresponding medium supplemented with 10% FBS for two weeks. Subsequently, the cells were fixed with 4% paraformaldehyde solution for 30 minutes before washed by PBS, and then stained with 0.3% crystal violet for another 15 minutes. Finally, the number of colonies was counted and analyzed.

**Assessment of IHC**

The staining of TFAP2C or TRIM29 was assessed blindly and independently by two pathologists. The signals were quantified based on the intensity and percentage of positively stained cells. The TFAP2C or TRIM29 staining intensity was scored 0 to 3 (0 = negative; 1 = weak; 2 = moderate; 3 = strong). The proportion of TFAP2C or TRIM29-positive stained cells was scored as: 1 (0%-25%), 2 (26%-50%), 3 (51%-75%) and 4 (76%-100%). The level of TFAP2C or TRIM29 staining was evaluated by immunoreactivity score (IRS), which is calculated by multiplying the scores of staining intensity and percentage.

**In situ hybridization (ISH)**

The RNAscope® probe targeting circIL4R was designed and synthesized by Advanced Cell Diagnostics. The expression of circIL4R in TMAs was detected by ISH using the RNAscope® 2.5 HD Reagent Kit-Red (Advanced Cell Diagnostics, Hayward, CA) according to the manufacturer’s instructions. The images of ISH staining were obtained by an Olympus microscope (Tokyo, Japan).

**Animal experiments**

The female BALB/c nude mice ranged from 6 to 8 weeks old were obtained from Beijing Vital River Laboratory Animal Technology Co., Ltd. (Beijing, China) and housed under specific pathogen-free conditions. CRC cells that stably interfere with circIL4R (Luc-sh-circIL4R) and non-specific control (Luc-shCtrl) were validated and prepared for use. To construct Xenograft tumorigenesis model, 200μl PBS containing groups of Luc-sh-circIL4R and Luc-shCtrl cells were injected subcutaneously into the flanks of mice (5×10^6^ cells) as shown in Fig 9a. The tumors volume (V) was calculated every 4 days by the following formula: V= (long axis× short axis^2^)/2. Four weeks after the injection, the mice were sacrificed, and the subcutaneous tumors were taken to calculate the volume and mass before used for HE and IHC staining. Similarly, to construct distant metastasis model, Groups of Luc-sh-circIL4R and Luc-shCtrl cells (1×10^6^ cells) were injected into the tail vein of athymic nude mice as shown in Fig 9g. The fluorescence intensity of lung metastasis was monitored by a Xenogen IVIS Spectrum (PerkinElmer, USA). Eight weeks later, the lungs of nude mice were dissected and the number of macroscopically visible metastasis nodules were counted and confirmed by HE staining. The Animal Care and Use Committee at Xuzhou Medical University provided the ethics approval statements for all the animal experiments in this study.
