# Supplementary figures and images for: CircIL4R activates the PI3K/AKT signaling pathway via the miR-761/TRIM29/PHLPP1 axis and promotes proliferation and metastasis in colorectal cancer
Source: Mol Cancer. 2021 Dec 18;20:167. doi: 10.1186/s12943-021-01474-9 (PMC8684286; doi:10.1186/s12943-021-01474-9)

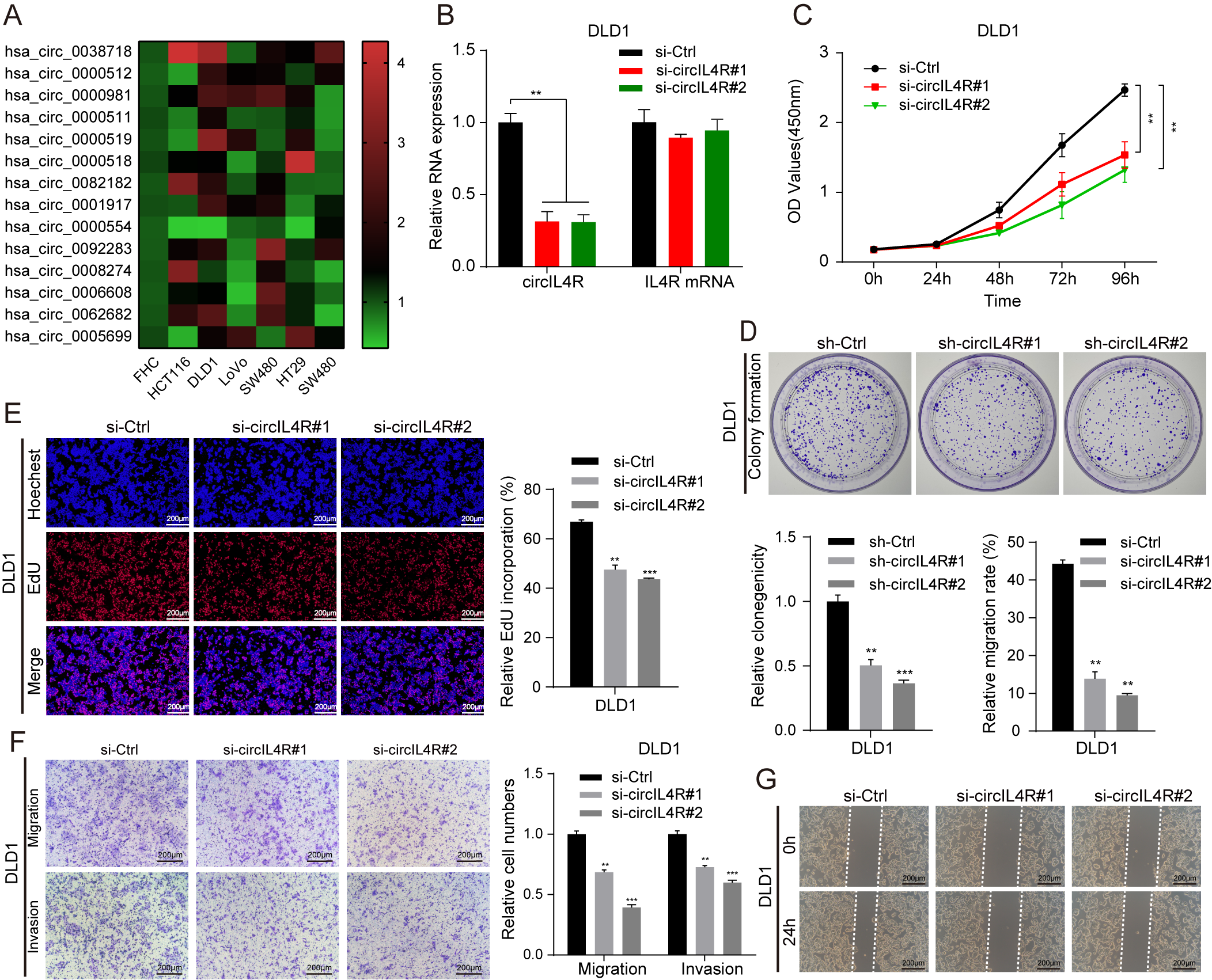

Supplement: Supplementary file 3 — Additional file 3: Figure S1. a. qRT-PCR validation of novel circRNAs expressed in different CRC cell lines and FHC cells; the circRNAs are clustered in a heatmap. b. qRT-PCR validation of circIL4R and IL4R mRNA expression in DLD1 cells transfected with siRNAs into DLD1 cells. c. The viability of DLD1 cells with circIL4R knockdown was detected by CCK-8 assays at the indicated time points. d. A colony formation assay was conducted to determine the proliferation of DLD1 cells stably transfected with sh-Ctrl or sh-circIL4R. e. An EdU assay was performed to assess the proliferation of DLD1 cells transfected with the indicated siRNAs. f and g. Representative images and quantification of Transwell and wound healing assays of DLD1 cells transfected with the indicated siRNAs. The data are presented as the means ± SD. *P < 0.05, **P < 0.01, ***P < 0.001. Figure S2. a-c. qRT-PCR validation of miR-761 expression in CRC cells transfected with miR-761 mimics or inhibitor into CRC cells. d-i. The CCK-8 and Transwell assays showed that the inhibitory effect of circIL4R knockdown on the proliferation, migration and invasion of HCT116 and DLD1 cells was reversed by miR-761 inhibitor, whereas the stimulatory effect of circIL4R overexpression on the proliferation, migration and invasion of LoVo cells was reversed by miR-761 mimics. j and k. Western blots showed that the reduction in p-AKT levels in HCT116 cells transfected with circIL4R siRNAs was reversed by miR-761 inhibitor, whereas the increase in p-AKT levels in LoVo cells transfected with circIL4R was reversed by miR-761 mimics. *P < 0.05, **P < 0.01, ***P < 0.001. Figure S3. a. TRIM29 expression was upregulated in CRC samples based on the TCGA COAD and READ databases. b and c. The transfection efficiency of the indicated TRIM29 siRNAs or overexpression plasmids was verified at the protein and mRNA level by western blot and qRT-PCR, respectively. d and e. qRT-PCR validation of circIL4R expression in HCT116 and DLD1 cells stably [file 12943_2021_1474_MOESM3_ESM.zip › Figure S1_ESM.tif]

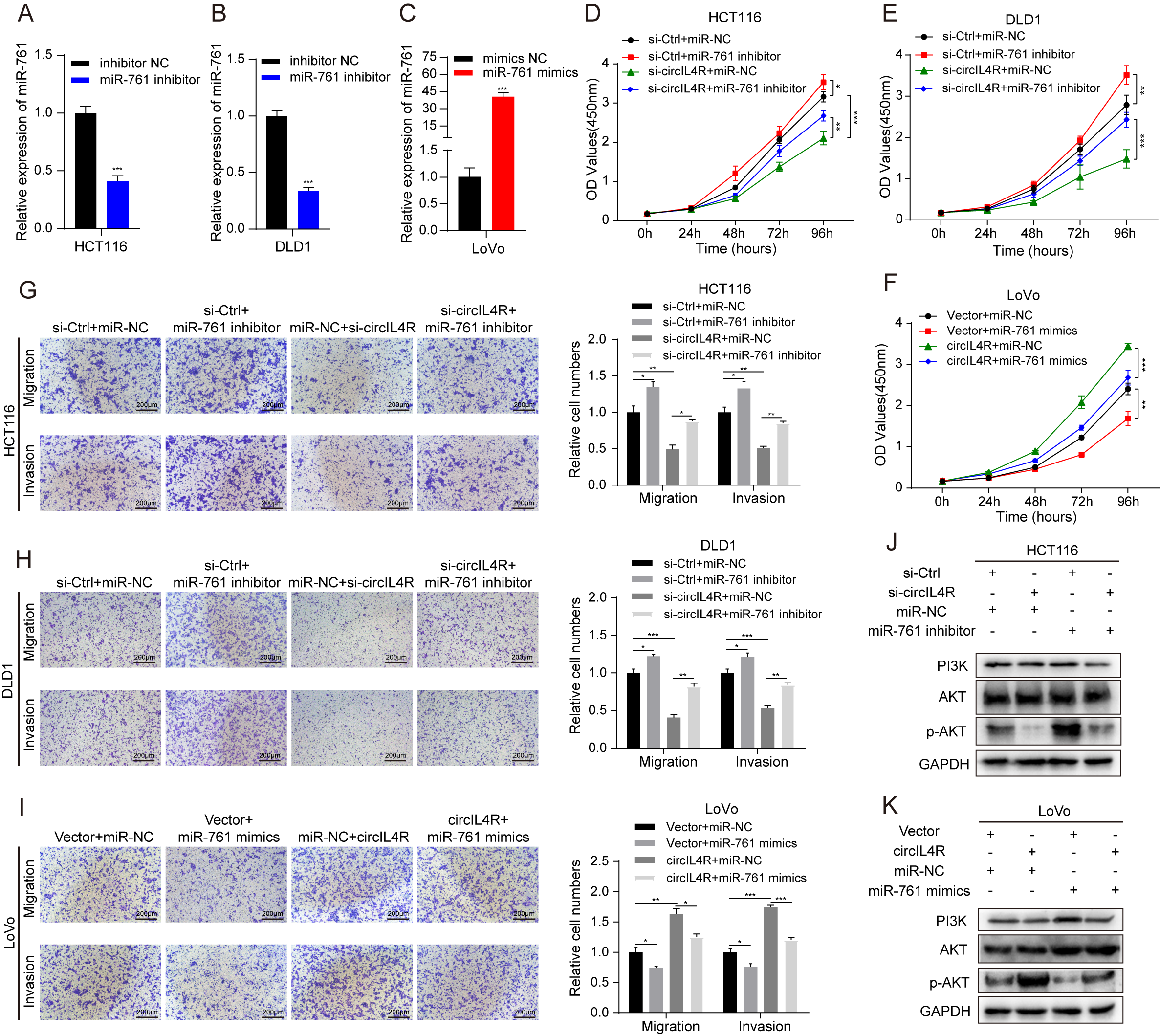

Supplement: Supplementary file 3 — Additional file 3: Figure S1. a. qRT-PCR validation of novel circRNAs expressed in different CRC cell lines and FHC cells; the circRNAs are clustered in a heatmap. b. qRT-PCR validation of circIL4R and IL4R mRNA expression in DLD1 cells transfected with siRNAs into DLD1 cells. c. The viability of DLD1 cells with circIL4R knockdown was detected by CCK-8 assays at the indicated time points. d. A colony formation assay was conducted to determine the proliferation of DLD1 cells stably transfected with sh-Ctrl or sh-circIL4R. e. An EdU assay was performed to assess the proliferation of DLD1 cells transfected with the indicated siRNAs. f and g. Representative images and quantification of Transwell and wound healing assays of DLD1 cells transfected with the indicated siRNAs. The data are presented as the means ± SD. *P < 0.05, **P < 0.01, ***P < 0.001. Figure S2. a-c. qRT-PCR validation of miR-761 expression in CRC cells transfected with miR-761 mimics or inhibitor into CRC cells. d-i. The CCK-8 and Transwell assays showed that the inhibitory effect of circIL4R knockdown on the proliferation, migration and invasion of HCT116 and DLD1 cells was reversed by miR-761 inhibitor, whereas the stimulatory effect of circIL4R overexpression on the proliferation, migration and invasion of LoVo cells was reversed by miR-761 mimics. j and k. Western blots showed that the reduction in p-AKT levels in HCT116 cells transfected with circIL4R siRNAs was reversed by miR-761 inhibitor, whereas the increase in p-AKT levels in LoVo cells transfected with circIL4R was reversed by miR-761 mimics. *P < 0.05, **P < 0.01, ***P < 0.001. Figure S3. a. TRIM29 expression was upregulated in CRC samples based on the TCGA COAD and READ databases. b and c. The transfection efficiency of the indicated TRIM29 siRNAs or overexpression plasmids was verified at the protein and mRNA level by western blot and qRT-PCR, respectively. d and e. qRT-PCR validation of circIL4R expression in HCT116 and DLD1 cells stably [file 12943_2021_1474_MOESM3_ESM.zip › Figure S2_ESM.tif]

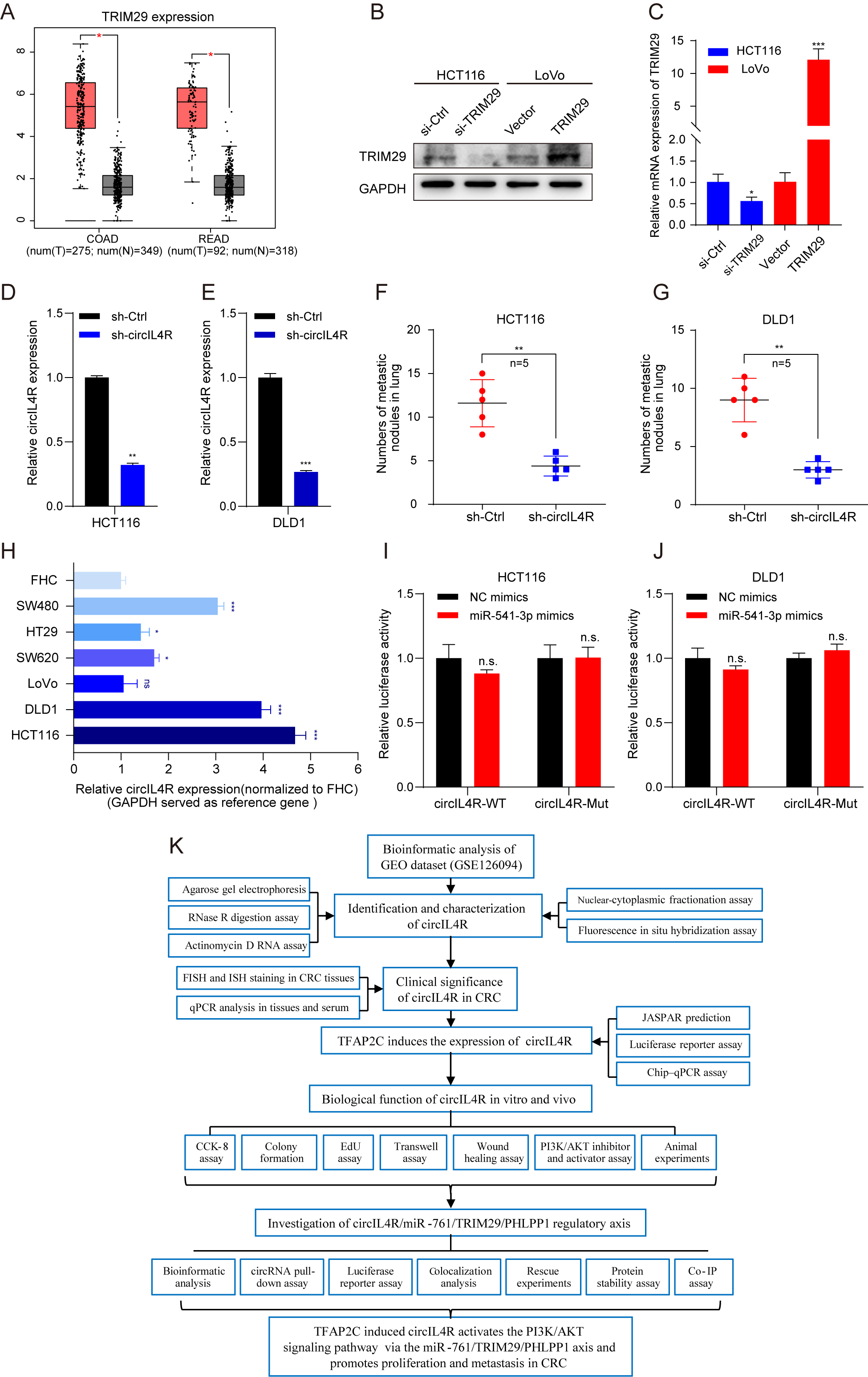

Supplement: Supplementary file 3 — Additional file 3: Figure S1. a. qRT-PCR validation of novel circRNAs expressed in different CRC cell lines and FHC cells; the circRNAs are clustered in a heatmap. b. qRT-PCR validation of circIL4R and IL4R mRNA expression in DLD1 cells transfected with siRNAs into DLD1 cells. c. The viability of DLD1 cells with circIL4R knockdown was detected by CCK-8 assays at the indicated time points. d. A colony formation assay was conducted to determine the proliferation of DLD1 cells stably transfected with sh-Ctrl or sh-circIL4R. e. An EdU assay was performed to assess the proliferation of DLD1 cells transfected with the indicated siRNAs. f and g. Representative images and quantification of Transwell and wound healing assays of DLD1 cells transfected with the indicated siRNAs. The data are presented as the means ± SD. *P < 0.05, **P < 0.01, ***P < 0.001. Figure S2. a-c. qRT-PCR validation of miR-761 expression in CRC cells transfected with miR-761 mimics or inhibitor into CRC cells. d-i. The CCK-8 and Transwell assays showed that the inhibitory effect of circIL4R knockdown on the proliferation, migration and invasion of HCT116 and DLD1 cells was reversed by miR-761 inhibitor, whereas the stimulatory effect of circIL4R overexpression on the proliferation, migration and invasion of LoVo cells was reversed by miR-761 mimics. j and k. Western blots showed that the reduction in p-AKT levels in HCT116 cells transfected with circIL4R siRNAs was reversed by miR-761 inhibitor, whereas the increase in p-AKT levels in LoVo cells transfected with circIL4R was reversed by miR-761 mimics. *P < 0.05, **P < 0.01, ***P < 0.001. Figure S3. a. TRIM29 expression was upregulated in CRC samples based on the TCGA COAD and READ databases. b and c. The transfection efficiency of the indicated TRIM29 siRNAs or overexpression plasmids was verified at the protein and mRNA level by western blot and qRT-PCR, respectively. d and e. qRT-PCR validation of circIL4R expression in HCT116 and DLD1 cells stably [file 12943_2021_1474_MOESM3_ESM.zip › Figure S3_ESM.tif]

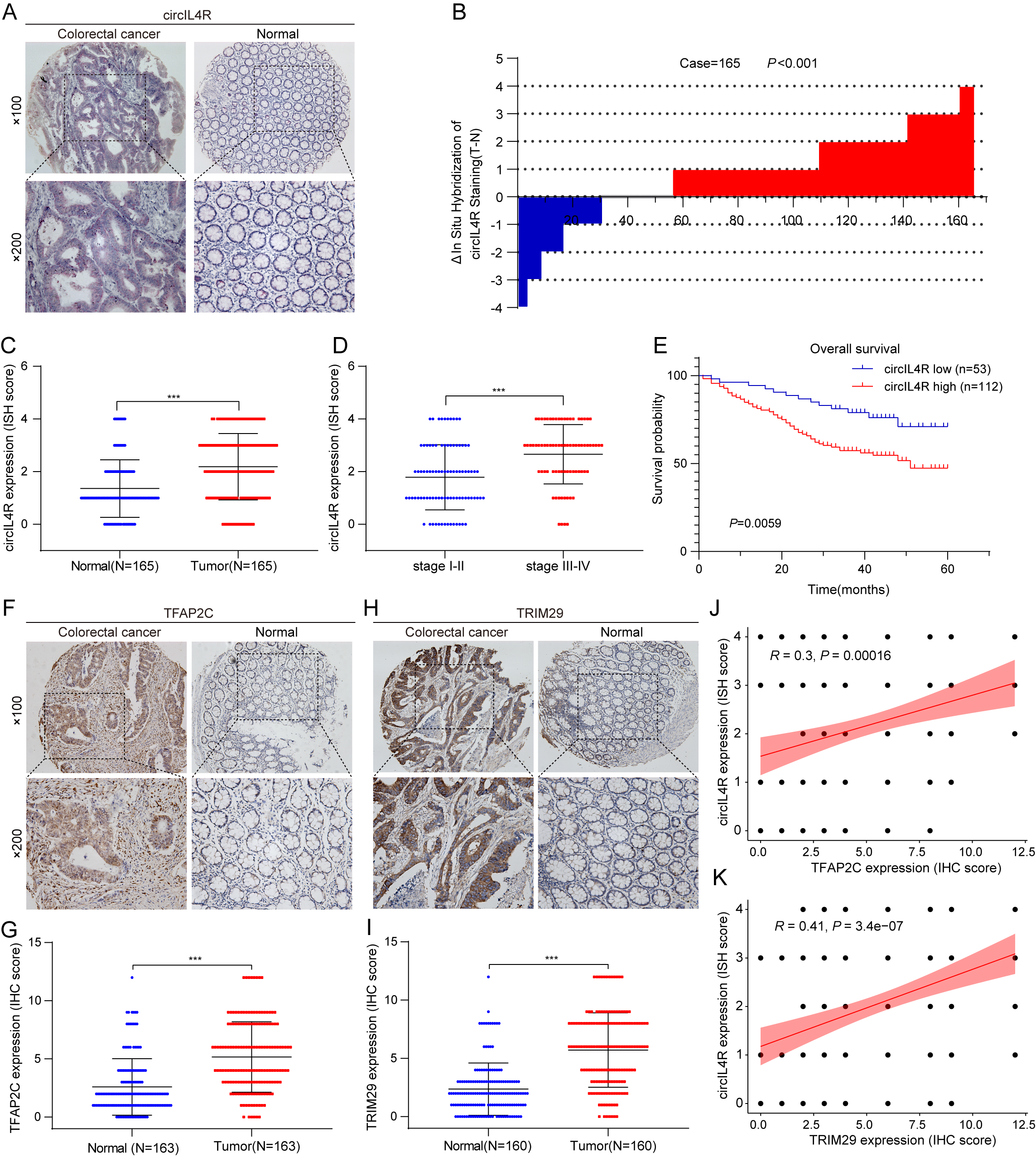

Supplement: Supplementary file 3 — Additional file 3: Figure S1. a. qRT-PCR validation of novel circRNAs expressed in different CRC cell lines and FHC cells; the circRNAs are clustered in a heatmap. b. qRT-PCR validation of circIL4R and IL4R mRNA expression in DLD1 cells transfected with siRNAs into DLD1 cells. c. The viability of DLD1 cells with circIL4R knockdown was detected by CCK-8 assays at the indicated time points. d. A colony formation assay was conducted to determine the proliferation of DLD1 cells stably transfected with sh-Ctrl or sh-circIL4R. e. An EdU assay was performed to assess the proliferation of DLD1 cells transfected with the indicated siRNAs. f and g. Representative images and quantification of Transwell and wound healing assays of DLD1 cells transfected with the indicated siRNAs. The data are presented as the means ± SD. *P < 0.05, **P < 0.01, ***P < 0.001. Figure S2. a-c. qRT-PCR validation of miR-761 expression in CRC cells transfected with miR-761 mimics or inhibitor into CRC cells. d-i. The CCK-8 and Transwell assays showed that the inhibitory effect of circIL4R knockdown on the proliferation, migration and invasion of HCT116 and DLD1 cells was reversed by miR-761 inhibitor, whereas the stimulatory effect of circIL4R overexpression on the proliferation, migration and invasion of LoVo cells was reversed by miR-761 mimics. j and k. Western blots showed that the reduction in p-AKT levels in HCT116 cells transfected with circIL4R siRNAs was reversed by miR-761 inhibitor, whereas the increase in p-AKT levels in LoVo cells transfected with circIL4R was reversed by miR-761 mimics. *P < 0.05, **P < 0.01, ***P < 0.001. Figure S3. a. TRIM29 expression was upregulated in CRC samples based on the TCGA COAD and READ databases. b and c. The transfection efficiency of the indicated TRIM29 siRNAs or overexpression plasmids was verified at the protein and mRNA level by western blot and qRT-PCR, respectively. d and e. qRT-PCR validation of circIL4R expression in HCT116 and DLD1 cells stably [file 12943_2021_1474_MOESM3_ESM.zip › Figure S4_ESM.tif]
