# Supplementary material for: CircIL4R activates the PI3K/AKT signaling pathway via the miR-761/TRIM29/PHLPP1 axis and promotes proliferation and metastasis in colorectal cancer
Source: Mol Cancer. 2021 Dec 18;20:167. doi: 10.1186/s12943-021-01474-9 (PMC8684286; doi:10.1186/s12943-021-01474-9)
Supplement: Supplementary file 1 — Additional file 1: Table S1. Clinical characteristics of 120 CRC samples used for qRT-PCR validation. Table S2. The sequences of siRNAs and shRNAs used in this study. Table S3. The sequences of primers used for qRT-PCR. Table S4. Relationship between circIL4R expression and clinicopathological features in fresh-frozen specimens. Table S5. Univariate Cox regression analysis of circIL4R expression and clinicopathologic variables predicting the survival of CRC patients. Table S6. Multivariate Cox regression analysis of circIL4R expression and clinicopathologic variables predicting the survival of CRC patients. Table S7. Prediction of miR-761 target genes based on four databases. Table S8. Relationship between circIL4R expression and clinicopathological features in TMAs specimens. [file 12943_2021_1474_MOESM1_ESM.zip › Table S2.doc]

| **siRNAs** | |
| --- | --- |
| si-Ctrl sense | UUCUCCGAACGUGUCACGUTT |
| si-Ctrl antisense | ACGUGACACGUUCGGAGAATT |
| si-circIL4R#1 sense | CUCUCCGAGUGCCUUGGCATT |
| si-circIL4R#1 antisense | UGCCAAGGCACUCGGAGAGTT |
| si-circIL4R#2 sense | CUGCUCUCCGAGUGCCUUGTT |
| si-circIL4R#2 antisense | CAAGGCACUCGGAGAGCAGTT |
| si-TFAP2C sense | GUAAACCAGUGGCAGAAUATT |
| si-TFAP2C antisense | UAUUCUGCCACUGGUUUACTT |
| si-TRIM29 sense | GUGCAUUGAUGAGCAAUUATT |
| si-TRIM29 antisense | UAAUUGCUCAUCAAUGCACTT |
| si-PHLPP1 sense | UUACCCAGUUCCAAAUUAUTT |
| si-PHLPP1 antisense | AUAAUUUGGAACUGGGUAATT |
| **shRNAs** |  |
| sh-Ctrl sense | TTCTCCGAACGTGTCACGT |
| sh-circIL4R#1 sense | CTCTCCGAGTGCCTTGGCA |
| sh-circIL4R#2 sense | CTGCTCTCCGAGTGCCTTG |
| **miR-761 mimics and inhibitor** | |
| mimics NC sense | UUGUACUACACAAAAGUACUG |
| miR-761 mimics sense | GCAGCAGGGUGAAACUGACACA |
| miR-761 mimics antisense | UGUCAGUUUCACCCUGCUGCUU |
| miR-541-3p mimics sense | UGGUGGGCACAGAAUCUGGACU |
| miR-541-3p mimics antisense | UCCAGAUUCUGUGCCCACCAUU |
| inhibitor NC sense | CAGUACUUUUGUGUAGUACAA |
| miR-761 inhibitor sense | UGUGUCAGUUUCACCCUGCUGC |

**Table S2.** The sequences of oligonucleotides and probes used in this study.
